# Coordinated regulation of chromatin modifiers reflects organised epigenetic programming in mouse oocytes

**DOI:** 10.1186/s13072-025-00583-9

**Published:** 2025-04-05

**Authors:** Chloe A. Edwards-Lee, Ellen G. Jarred, Patrick S. Western

**Affiliations:** Centre for Reproductive Health, Department of Molecular and Translational Science, Hudson Institute of Medical Research, Monash University, Clayton, VIC Australia

**Keywords:** Epigenetic programming, Maternal germline, Oocytes, Histone modifications, DNA methylation, PRC2, EED, SETD2

## Abstract

**Background:**

Epigenetic modifications provide mechanisms for influencing gene expression, regulating cell differentiation and maintaining long-term memory of cellular identity and function. As oocytes transmit epigenetic information to offspring, correct establishment of the oocyte epigenome is important for normal offspring development. Oocyte epigenetic programming is highly complex, involving a range of epigenetic modifiers which interact to establish a specific distribution of DNA methylation and histone modifications. Disruptions to oocyte epigenetic programming can alter epigenetic memory and prevent normal developmental outcomes in the next generation. Therefore, it is critical that we further our understanding of the interdependent relationships between various epigenetic modifiers and modifications during oogenesis.

**Results:**

In this study we investigated the spatial and temporal distribution of a range of epigenetic modifiers and modifications in growing oocytes of primordial to antral follicles. We provide comprehensive immunofluorescent profiles of SETD2, H3K36me3, KDM6A, RBBP7, H3K27me3, DNMT3A and DNMT3L and compare these profiles to our previously published profiles of the Polycomb Repressive Complex 2 components EED, EZH2 and SUZ12 in growing oocytes of wildtype mice. In addition, we examined the nuclear levels and spatial distribution of these epigenetic modifiers and modifications in oocytes that lacked the essential Polycomb Repressive Complex 2 subunit, EED. Notably, histone remodelling in primary-secondary follicle oocytes preceded upregulation of DNMT3A and DNMT3L in secondary-antral follicle oocytes. Moreover, loss of EED and H3K27me3 led to significantly increased levels of the H3K36me3 methyltransferase SETD2 during early-mid oocyte growth, although the average levels of H3K36me3 were unchanged.

**Conclusions:**

Overall, these data demonstrate that oocyte epigenetic programming is a highly ordered process, with histone remodelling in early growing oocytes preceding *de novo* DNA methylation in secondary-antral follicle oocytes. These results indicate that tight temporal and spatial regulation of histone modifiers and modifications is essential to ensure correct establishment of the unique epigenome present in fully grown oocytes. Further understanding of the temporal and spatial relationships between different epigenetic modifications and how they interact is essential for understanding how germline epigenetic programming affects inheritance and offspring development in mammals, including humans.

**Supplementary Information:**

The online version contains supplementary material available at 10.1186/s13072-025-00583-9.

## Introduction

Epigenetic modifications, such as DNA methylation and histone modifications, regulate chromatin accessibility and recruitment of transcription factors to modulate gene transcription. Establishment of cell type specific epigenetic combinations underpins cell specification, and the maintenance of cell specific epigenetic patterns through cell divisions sustains long term cellular identity and function [1]. Disruption of epigenetic information can lead to disease, such as cancers, and developmental, metabolic and behavioural disorders [2–4].

In addition to mitotic heritability, epigenetic modifications can also be transmitted through meiosis in germline cells to be inherited by offspring at fertilisation [5]. The best studied mechanism of intergenerational epigenetic inheritance is genomic imprinting, which involves differential establishment of sex-specific DNA methylation in the maternal or paternal germlines, or Histone 3 lysine 27 trimethylation (H3K27me3) in the maternal germline, and subsequent parent-specific gene expression in offspring [6, 7].

Early germ cells undergo extensive epigenetic reprogramming which removes maternal and paternal imprints inherited from the previous generation. This allows germ cells to establish new sex-specific imprints in oocytes or sperm and transmit these parent-specific epigenetic modifications to offspring [8, 9]. As altered epigenetic programming in the germline can disrupt offspring development, it is essential that epigenetic information is correctly established in oocytes and sperm [10–14].

In females, maternal epigenetic programming occurs postnatally as oocytes grow within primary to antral follicles. During the later stages of oocyte development in antral follicles, mammalian oocytes are characterised by chromatin condensation associated with the germinal vesicle (GV) [15]. In mice, this includes formation of heterochromatin that delineates the surrounded nucleolus (SN) GV stage at which time the oocyte epigenome has largely been established and the fully grown oocyte has a unique epigenome with a combination of active and repressive epigenetic modifications [16, 17]. Establishment of H3K36me3 promotes DNA methylation, and these modifications are found together in actively transcribed regions in oocytes [18]. H3K27me3 marks repressed chromatin and occupies different regions of the oocyte genome to H3K36me3 and DNA methylation. Loss of the H3K36 methyltransferase SETD2 (SET domain containing 2, histone lysine methyltransferase) in mice permits ectopic spreading of H3K27me3, indicating that H3K36me3 is antagonistic to H3K27me3 and demarcates active genomic regions in fully grown oocytes [18]. In contrast, H3K27me3 is found in broad peaks that are established during early oocyte growth and is required for repression of a wide range of genes, many of which are involved in cell differentiation and fetal development [19, 20]. These examples highlight the complexity of oocyte epigenetic programming and that properly ordered arrangement of epigenetic enzymes and the modifications they establish in growing oocytes is essential.

PRC2 (Polycomb Repressive Complex 2) is a highly conserved epigenetic modifying complex composed of core subunits EED (Embryonic Ectoderm Development), EZH1/2 (Enhancer of Zeste 1 or 2), and SUZ12 (Suppressor of Zeste 12) which associate with RBBP4/7 (Retinoblastoma Binding Protein 4 or 7) to mediate H3K27 histone methyltransferase activity [21–23]. EED, EZH2, and SUZ12 are transiently detected together in primary-secondary follicle oocytes and *Eed* is required for H3K27me3 establishment and repression of a range of developmental genes in oocytes [20]. Oocyte-specific deletion of *Eed* also causes loss of H3K27me3-dependent (non-canonical) imprinting and biallelic expression of non-canonically imprinted genes in offspring [24, 25]. Embryos from oocytes with *Eed* deleted are initially characterised by developmental delay and male-biased lethality [14, 24, 26], however this growth restriction is followed by placental hyperplasia, fetal catch-up growth and postnatal offspring overgrowth [10, 14, 27]. Loss of maternal H3K27me3 imprinting contributes to these phenotypes as deletion of maternal *Slc38a4* (*Solute carrier family 38 member 4*) or an imprinted cluster of microRNAs within *Sfmbt2* (*Scm like with fur mbt domains 2*) in addition to *Eed* in oocytes rescued placental hyperplasia in offspring [27]. Similarly, deletion of maternal *Xist* (*X-inactive specific transcript*) rescued male biased lethality, but only partially rescued the growth restriction observed in offspring [27]. This implies that other mechanism(s) related to loss of H3K27me3 contribute to phenotypic outcomes in offspring [20].

One possibility is that H3K27me3 prevents establishment of H3K36me3/DNA methylation within growing oocytes, ensuring that an appropriate epigenetic signature is inherited and supports normal offspring development [20]. SETD2 is required for proper oocyte epigenetic programming, including correct establishment of H3K36me3 and DNA methylation and preventing spread of H3K27me3 into inappropriate genomic regions [18].

Despite the importance of temporal and spatial regulation of maternal epigenetic programming, the temporal and spatial distribution of enzymes and epigenetic modifications that mediate these processes are poorly described during oocyte growth. This study provides comprehensive immunofluorescent profiles of RBBP7, SETD2, KDM6A (Lysine demethylase 6 A), DNMT3A (DNA methyltransferase 3 alpha), DNMT3L (DNA methyltransferase 3 like), H3K36me3, and H3K27me3 throughout oocyte growth in wildtype mice, relative to the PRC2 subunits EED, EZH2 and SUZ12. Our data support a model in which oocyte epigenetic programming is a highly ordered process, with histone modification remodelling preceding expression of DNA methyltransferases. Moreover, we investigate how the average nuclear levels and localisation of these epigenetic modifiers and modifications are impacted by oocyte-specific loss of *Eed* and H3K27me3 depletion within growing oocytes.

## Methods

### Mouse strains, animal housing, breeding and ethics

Mice were housed at Monash Medical Centre Animal Facility using a 12-hour light/dark cycle, temperature maintained at 21–23˚C, controlled humidity, and food and water available *ad libitum* as previously described [10]. All animal breeding and experimental work was undertaken in accordance with Monash University Animal Ethics Committee approvals MMCB/2021/30/BC and MMCB/2020/37. Mice were obtained from the following sources: *Zp3Cre* mice C57BL/6-Tg 93knw/J; Jackson Labs line 003651, constructed and shared by Professor Barbara Knowles [28], *Eed* floxed mice (*Eed*^fl/fl^) B6; 129S1-*Eed*tm1Sho/J; Jackson Labs line 0022727; constructed and shared by Professor Stuart Orkin [29]. The *Eed* line was backcrossed to a pure C57BL6/J and shared with us by Associate Professor Rhys Allen and Professor Marnie Blewitt, Walter and Eliza Hall Institute for Medical Research, Melbourne.

### Genotyping

Ear punch tissue was collected at weaning and genotyping performed by Transnetyx (Cordova, TN) using quantitative PCR assays (details available upon request) designed for each gene as described previously [10].

### Tissue collection, fixation and embedding

Ovaries for immunofluorescence (IF) were collected from adult female mice between 7 and 12 weeks old, fixed in 4% paraformaldehyde (PFA) overnight at 4˚C, processed through a standard ethanol series and embedded in paraffin blocks by Monash Histology Platform.

### Immunofluorescence

Five micrometre (μm) sections were cut in a compound series and transferred to Superfrost™ Plus slides (Thermo Fisher) followed by DAKO citrate antigen retrieval at 98˚C for 30 min. Slides were blocked for non-specific binding in PBS containing 5% BSA and 10% donkey serum for 1 h at room temperature (RT). Blocking solution was replaced with PBS containing 0.1% Triton X-100, 1% BSA and appropriately diluted primary antibodies (Table 1), and incubated overnight at 4˚C. Slides were washed with PBS, then incubated with PBS containing 0.1% Triton X-100, 1% BSA and appropriately diluted secondary antibodies (Table 2) for 1 h in the dark at RT. For control slides, primary antibody was omitted, and only secondary antibody was included. Slides were again washed with PBS, rinsed in distilled H2O, then mounted in DAPI Prolong™ Gold (Thermo Fisher) and left to dry overnight. Slides were imaged at 40x using the Olympus VS120 or VS200 Slide Scanner by a Monash Histology Platform employee who is dedicated to running these instruments. Acquisition settings were optimised for each antibody to ensure signal capture at peak levels and across the full dynamic range for the instrument without signal saturation. All slides for each antibody were stained and imaged together on the same instrument, with identical scanner settings used for all samples and genotypes for each individual antibody, ensuring that staining conditions, scanning levels and data acquisition were controlled as precisely as possible. Analysis and quantification were performed using QuPath Bioimage Analysis Software [30]. All follicles were classified according to Pederson classification [31]. The oocyte nucleus was annotated as the region of interest based on Lamin B1 staining, which detects the nuclear membrane. Average nuclear staining intensity within this region was calculated, and a measurement for non-specific background staining in the oocyte cytoplasm was subtracted specific to follicle stage. For follicle stage comparisons in WT growing oocytes, raw values were plotted on the graph as average intensity, with no normalisation performed. For comparisons between *Eed*-wt, *Eed*-het, and *Eed*-hom oocytes, the average intensity of *Eed*-wt was set to 1.0 for each follicle stage, and the *Eed*-het and *Eed*-hom values for the same follicle stage were normalised to *Eed*-wt and values displayed as relative intensity between genotypes. Primary antibodies used to detect epigenetic modifiers or modifications were chosen based on their previous use in other studies and their validation by the supplier using cells or tissues in which the target was deleted (knockout) or knocked down (DNMT3A, DNMT3L, RBBP7, SETD2, H3K36me3) or in cells that have lost the target (KDM6A), or in our own studies (H3K27me3 in EED KO mice and cells [10, 11, 20]; Table 1).

**Table 1:** Primary antibodies used for indirect immunofluorescence

Antibody	Species	Dilution	Validation	Supplier
DNMT3A	Rabbit	1:200	Knockout validated	Abcam, ab307503
DNMT3L	Rabbit	1:200	Knockdown validated	Cell Signaling Technology, 13451S
H3K27me3	Rabbit	1:800	Validated in our own studies (10, 11, 20)	Cell Signaling Technology, 9733S
H3K36me3	Rabbit	1:1000	Validated in A498 cells with SETD2 mutation	Cell Signaling Technology, 4909S
RBBP7	Rabbit	1:500	Knockout validated	Abcam, ab259957
SETD2	Rabbit	1:500	Knockdown validated	Cell Signaling Technology, 89680S
KDM6A	Rabbit	1:400	Validated in THP-1 cells with loss of KDM6A	Cell Signaling Technology, 33510S
Lamin B1 (marks nuclear membrane)	Goat	1:500		Santa Cruz Biotechnology, SC-6217

**Table 2:** Secondary antibodies used for indirect immunofluorescence

Antibody	Species	Dilution	Supplier
Goat 488	Donkey	1:500	Thermo Fisher, A11055
Rabbit 555	Donkey	1:500	Thermo Fisher, A31572

### RNA-sequencing data analyses

GV oocyte RNA-sequencing data from Jarred et al. 2022 ([20]; GEO accession number GSE193582) was analysed to assess transcript per million of genes encoding PRC2.1 and PRC2.2 accessory proteins, and H3K27me3 demethylases relative to one another in *Eed-wt* samples and between *Eed*-wt, *Eed*-het, and *Eed*-hom samples.

### Statistical analyses

As appropriate, one-way ANOVA plus Tukey’s multiple comparisons test, or a Kruskal-Wallis with Dunn’s multiple comparisons test were performed. Relevant information is stated in figure legends. GraphPad Prism (Version 9.3.1) was utilised for all statistical analyses and to graph data.

## Results

### SETD2 levels increased during primary-secondary follicle stages of oocyte growth

**Fig. 1:**
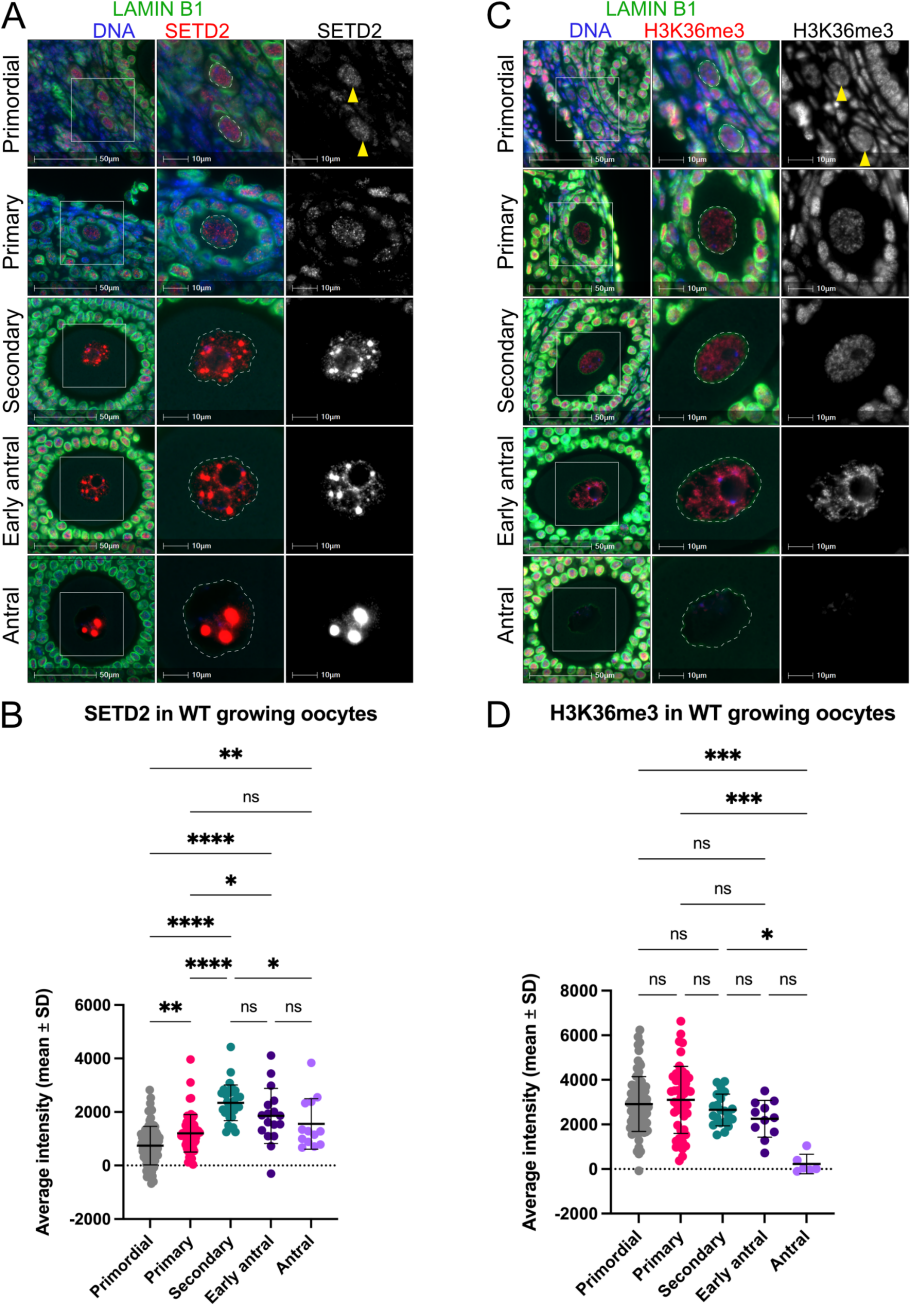
SETD2 and H3K36me3 peak in early growing oocytes before declining during late oocyte growth. (**A**,** C**) Representative IF images showing SETD2 (A) and H3K36me3 (C) (red, grey single channel) in primordial, primary, secondary, early antral and antral follicle oocytes from wildtype adult mouse ovaries. The oocyte nucleus is defined by Lamin B1 (green, dashed lines), and DNA is shown by DAPI (blue). White boxes indicate regions shown at higher power in images on the right. Yellow arrowheads denote primordial follicle oocyte nuclei. Images are representative of multiple planes in both ovaries from three biological replicates. Scale bars represent 50 μm (left), and 10 μm (middle, right). (**B**,** D**) Quantification of SETD2 (B) and H3K36me3 (D) within oocyte nuclei of primordial, primary, secondary, early antral and antral follicles from wildtype adult mouse ovaries. Values represent average nuclear staining intensity with average cytoplasmic fluorescence removed to correct for non-specific background staining. Error bars represent mean ± SD. (**B**) ns: not significant, *P < 0.05, **P < 0.01, ****P < 0.0001, one-way ANOVA plus Tukey’s multiple comparisons test. *N* = 94 primordial follicle oocytes, *N* = 56 primary follicle oocytes, *N* = 32 secondary follicle oocytes, *N* = 17 early antral follicle oocytes, and *N* = 13 antral follicle oocytes, in total from three biological replicates. (**D**) ns: not significant, *P < 0.05, ***P < 0.001, Kruskal-Wallis test with Dunn’s multiple comparisons test. *N* = 82 primordial follicle oocytes, *N* = 47 primary follicle oocytes, *N* = 21 secondary follicle oocytes, *N* = 11 early antral follicle oocytes, and *N* = 6 antral follicle oocytes, in total from three biological replicates

SETD2 was detected in the nucleus at all stages of oocyte growth, however levels clearly increased in primary-secondary follicle oocytes. In primordial follicle oocytes, SETD2 was detected with weak intensity broadly throughout the nucleus (Fig. 1A). In primary follicle oocytes, SETD2 was detected in a speckled pattern and at stronger intensity than that observed in primordial follicle oocytes (Fig. 1A). In secondary follicle oocytes, SETD2 was dispersed throughout the nucleus, but often showed distinct SETD2 foci amidst diffuse SETD2 staining (Fig. 1A). Notably, there was a significant increase in average SETD2 intensity in oocyte nuclei within primordial to primary follicle oocytes (*P* < 0.01, Fig. 1B) and a further increase from primary to secondary follicle oocytes with SETD2 levels peaking in secondary stage follicle oocytes (*P* < 0.0001, Fig. 1B). Following the increase in primary-secondary stage, early antral follicle oocytes displayed similar staining to secondary follicles, with distinct foci visible (Fig. 1A). However, while average SETD2 staining intensity remained relatively high in early antral and antral follicle oocytes compared to primary stage oocytes, the average intensity of SETD2 significantly decreased in antral compared to secondary follicle oocytes (*P* < 0.05, Fig. 1B, Additional File 1: Fig. S1A) and the signal became increasingly localised to specific sub-nuclear regions as oocyte growth progressed (Fig. 1A). Notably, this localisation varied from a single intense spot of SETD2 staining to a larger number of smaller intense foci (> 5).

### H3K36me3 staining was widespread throughout the nucleus for most of oocyte growth before substantially decreasing in antral follicle oocytes

SETD2 is essential for catalysing H3K36me3. In primordial follicle oocytes, H3K36me3 was already broadly detected throughout the nucleus (Fig. 1C). While SETD2 levels increased in primary-secondary follicle oocytes, global H3K36me3 levels were consistent across early oocyte growth (Fig. 1B and D). H3K36me3 staining intensity and pattern was similar in primordial, primary and secondary follicle oocytes, with uniform, slightly speckled staining throughout the oocyte nucleus (Fig. 1C). By early antral follicle stage, H3K36me3 was visible as small foci in more concentrated regions of the nucleus, with more intense H3K36me3 staining surrounding the nucleolus in many oocytes (Fig. 1C). Average H3K36me3 levels within oocyte nuclei did not significantly change from primordial, to primary, secondary, and early antral follicles (Fig. 1D). While SETD2 was still detected in antral follicle oocytes, H3K36me3 staining intensity substantially decreased (Fig. 1A, C). In some antral follicle oocytes, H3K36me3 was detected at low levels in localised regions of the nucleus, but in other oocytes H3K36me3 was not detected at all. While there was no significant decrease from secondary to antral stage when all antral follicle oocytes were considered together, separation into early antral and antral follicle stages revealed a significant decrease of H3K36me3 in late-stage antral follicles (*P* < 0.05, Fig. 1D; Additional File 1: Fig. S1B).

### RBBP7 staining substantially increased in secondary follicle oocytes and remained strong in early antral and antral follicle stages

**Fig. 2:**
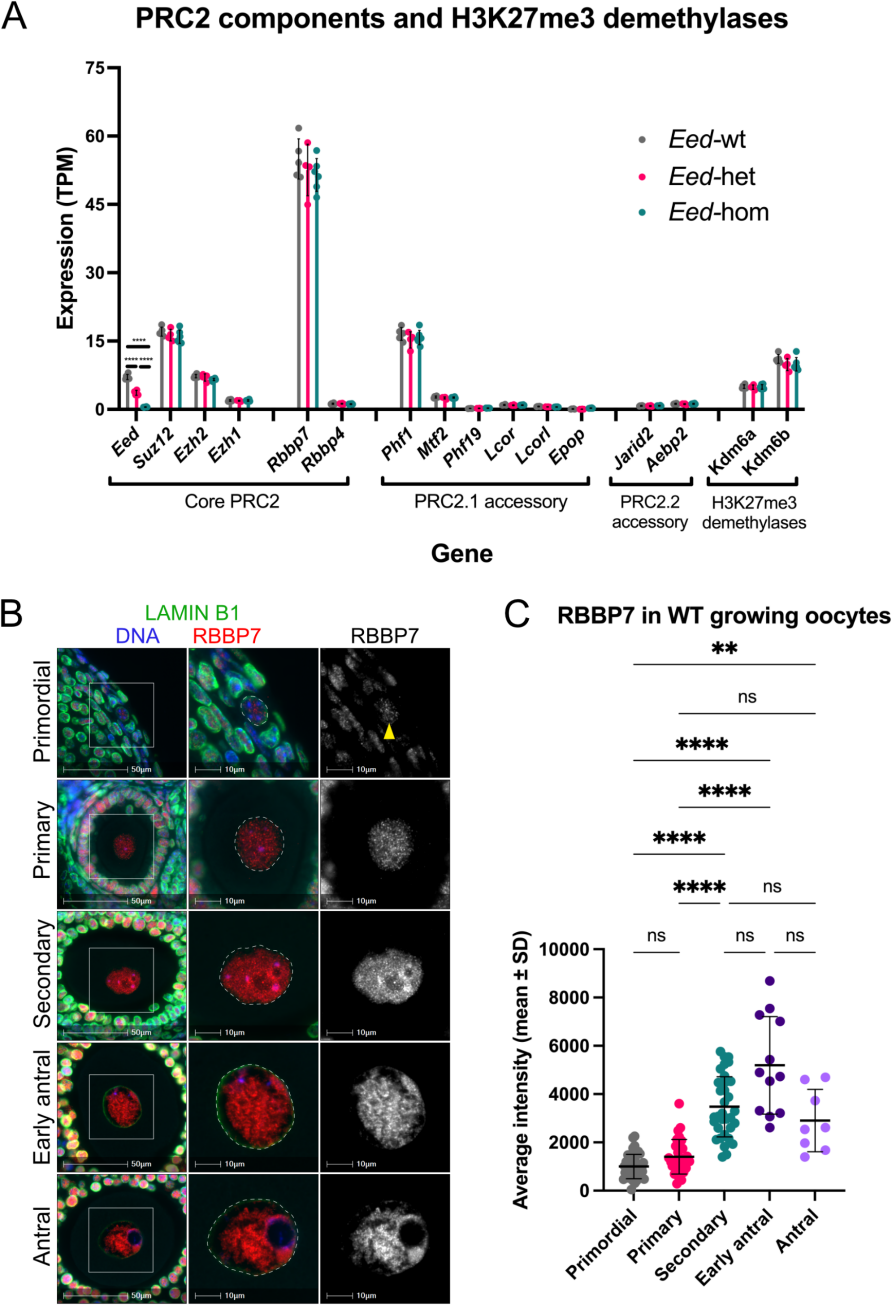
RBBP7 is present throughout oocyte growth but substantially increases in secondary-antral follicle oocytes. (**A**) Transcriptional levels of PRC2 core and PRC2.1 and PRC2.2 accessory components, and H3K27me3 demethylases in *Eed*-wt, *Eed*-het, and *Eed*-hom oocytes. Data is displayed as mean transcripts per million reads (TPM). Error bars represent mean ± SD. ****P < 0.0001, one-way ANOVA plus Tukey’s multiple comparisons test. RNA-seq data is from Jarred et al. 2022 ([20]; GEO accession number GSE193582). (**B**) Representative IF images showing RBBP7 (red, grey single channel) in primordial, primary, secondary, early antral and antral follicle oocytes from wildtype adult mouse ovaries. The oocyte nucleus is defined by Lamin B1 (green, dashed lines), and DNA is shown by DAPI (blue). White boxes indicate regions shown at higher power in images on the right. Yellow arrowhead denotes primordial follicle oocyte nucleus. Images are representative of multiple planes in both ovaries from two biological replicates. Scale bars represent 50 μm (left), and 10 μm (middle, right). (**C**) Quantification of RBBP7 within oocyte nuclei of primordial, primary, secondary, early antral and antral follicles from wildtype adult mouse ovaries. Values represent average nuclear staining intensity with average cytoplasmic fluorescence removed to correct for non-specific background staining. Error bars represent mean ± SD. ns: not significant, **P < 0.01, ****P < 0.0001, Kruskal-Wallis test with Dunn’s multiple comparisons test. *N* = 41 primordial follicle oocytes, *N* = 30 primary follicle oocytes, *N* = 35 secondary follicle oocytes, *N* = 12 early antral follicle oocytes, and *N* = 8 antral follicle oocytes, in total from two biological replicates

We previously reported the transcriptional levels of core PRC2 components *Eed*,* Ezh2* and *Suz12* in GV oocytes and the distribution of EED, EZH2, and SUZ12 during oocyte growth and maturation [20]. However, distribution of the essential co-factors RBBP4/7 and other PRC2 accessory proteins have not been reported. Analysis of RNA-sequencing data from GV oocytes revealed that *Rbbp7* and *Phf1* were transcribed at high levels relative to other core and accessory PRC2 components and that deletion of *Eed* did not affect transcription of any of the core and accessory PRC2 components analysed (Fig. 2A) [20]. Immunofluorescence revealed that RBBP7 was detected within the nucleus of primordial to antral follicle oocytes (Fig. 2B). In both primordial and primary follicle oocytes, RBBP7 was detected within the nucleus in a broad, speckled pattern (Fig. 2B). Nuclear RBBP7 levels differed between individual primordial and primary follicle oocytes, ranging from a level similar to background staining to strong expression that was also characterised by the presence of small foci. There was no significant change in average nuclear RBBP7 levels detected in primordial to primary follicle oocytes. RBBP7 was detected in a speckled pattern throughout the nucleus of secondary follicle oocytes, with substantially higher staining intensity than primary follicle stage oocytes (*P* < 0.0001, Fig. 2B, C). Furthermore, in secondary follicle oocytes, several RBBP7 foci were evident and were often closely colocalised with relatively intense DAPI foci indicating that RBBP7 may be increased in heterochromatic regions (Fig. 2B). RBBP7 staining remained strong during late oocyte growth, and there was no significant change in levels between secondary to antral follicles (Fig. 2C, Additional File 1: Fig. S1C). In early antral and antral follicle oocytes, RBBP7 was slightly more localised in intense foci, with weaker or no staining often in regions of the nuclear periphery, and a lack of staining in the nucleolus (Fig. 2B).

### H3K27me3 levels peaked during the early stages of oocyte growth

**Fig. 3:**
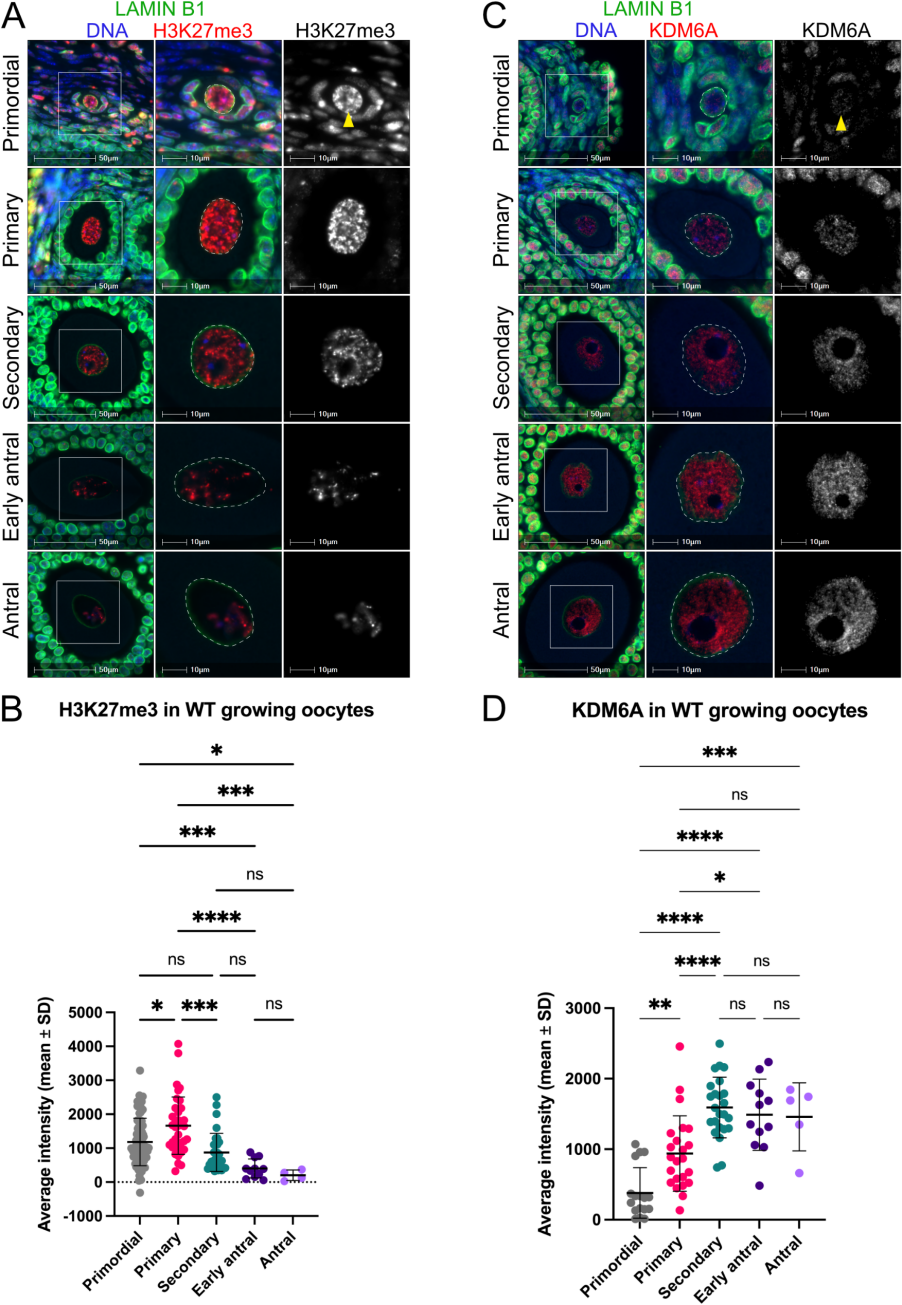
H3K27me3 peaks in primary follicle oocytes before declining coincident with increasing KDM6A in primary-secondary stage. (**A**,** C**) Representative IF images showing H3K27me3 (A) and KDM6A (C) (red, grey single channel) in primordial, primary, secondary, early antral and antral follicle oocytes from wildtype adult mouse ovaries. Images are representative of multiple planes in both ovaries from two to three biological replicates. The oocyte nucleus is defined by Lamin B1 (green, dashed lines), and DNA is shown by DAPI (blue). White boxes indicate regions shown at higher power in images on the right. Yellow arrowheads denote primordial follicle oocyte nuclei. Scale bars represent 50 μm (left), and 10 μm (middle, right). (**B**,** D**) Quantification of H3K27me3 (B) and KDM6A (D) within oocyte nuclei of primordial, primary, secondary, early antral and antral follicles from wildtype adult mouse ovaries. Values represent average nuclear staining intensity with average cytoplasmic fluorescence removed to correct for non-specific background staining. Error bars represent mean ± SD. (B) ns: not significant, *P < 0.05, ***P < 0.001, ****P < 0.0001, Kruskal-Wallis test with Dunn’s multiple comparisons test. *N* = 84 primordial follicle oocytes, *N* = 37 primary follicle oocytes, *N* = 32 secondary follicle oocytes, *N* = 11 early antral follicle oocytes, and *N* = 4 antral follicle oocytes, in total from three biological replicates. (**D**) Quantification of KDM6A within oocyte nuclei of primordial, primary, secondary, early antral and antral follicles from wildtype adult mouse ovaries. ns: not significant, *P < 0.05, **P < 0.01, ***P < 0.001, ****P < 0.0001, one-way ANOVA plus Tukey’s multiple comparisons test. *N* = 17 primordial follicle oocytes, *N* = 23 primary follicle oocytes, *N* = 24 secondary follicle oocytes, *N* = 12 early antral follicle oocytes, and *N* = 5 antral follicle oocytes, in total from two biological replicates

The PRC2 core components EED, EZH2, and SUZ12 are detected in oocytes of primary-secondary follicles and mediate deposition of H3K27me3 during this window of oocyte growth [20]. However, a detailed description of H3K27me3 staining distribution throughout oocyte growth has not been reported. In primordial follicle oocytes, H3K27me3 staining was distributed throughout the nucleus and varied from low level diffuse staining to more intense foci (Fig. 3A). A similar speckled nuclear staining pattern was evident in primary follicle oocytes, with many foci distributed throughout the nucleus (Fig. 3A). H3K27me3 levels increased in primary compared to primordial follicle oocytes (*P* < 0.05, Fig. 3B). Secondary follicle oocytes contained more diffuse staining and a lower total nuclear level of H3K27me3 than primary follicle oocytes (*P* < 0.001, Fig. 3A, B). While not quantified, there also appeared to be fewer foci in secondary than in primary follicle oocyte nuclei. H3K27me3 was initially broadly detected within the nucleus but was increasingly localised to sub-nuclear regions during later oocyte growth. In early antral follicle oocytes, H3K27me3 was primarily confined to a relatively small number of foci, a pattern that was further accentuated by the antral follicle stage (Fig. 3A). Overall, while H3K27me3 levels increased during early oocyte growth and peaked in primary stage oocyte nuclei, total nuclear H3K27me3 levels subsequently decreased in secondary follicle oocytes and remained low in later oocyte growth (Fig. 3B). H3K27me3 levels significantly decreased from secondary to antral stage when all antral follicles were analysed together (*P* < 0.05, Additional File 1: Fig. S1D).

### The H3K27me3 demethylase, KDM6A was increased during later stages of oocyte growth

KDM6A (also known as UTX) and KDM6B (also known as JMJD3) both mediate demethylation of H3K27me3 through their Jumonji C (JmjC) domain [32]. Quantification of *Kdm6a* and *Kdm6b* transcription in GV oocytes revealed that *Kdm6b* levels were higher than *Kdm6a* (Fig. 2A). While KDM6B antibody staining was unsuccessful, KDM6A was detected in a broadly distributed speckled pattern throughout the nucleus of primary-antral stage oocytes (Fig. 3C). Although KDM6A was very low or not detected in primordial follicle oocytes, levels increased notably in primary stage follicles (*P* < 0.01, Fig. 3C, D). Moreover, KDM6A levels increased further in nuclei of secondary follicle oocytes (*P* < 0.0001, Fig. 3D) in parallel with decreasing H3K27me3 (Fig. 3B). KDM6A was then maintained at higher levels in early antral and antral follicles than in primary or primordial stage oocytes, suggesting a possible role in demethylating H3K27 during the later stages of oocyte growth (Fig. 3C, D, Additional File 1: Fig. S1E).

### *De novo* DNA methyltransferases are detected in oocyte nuclei from the secondary follicle stage

**Fig. 4:**
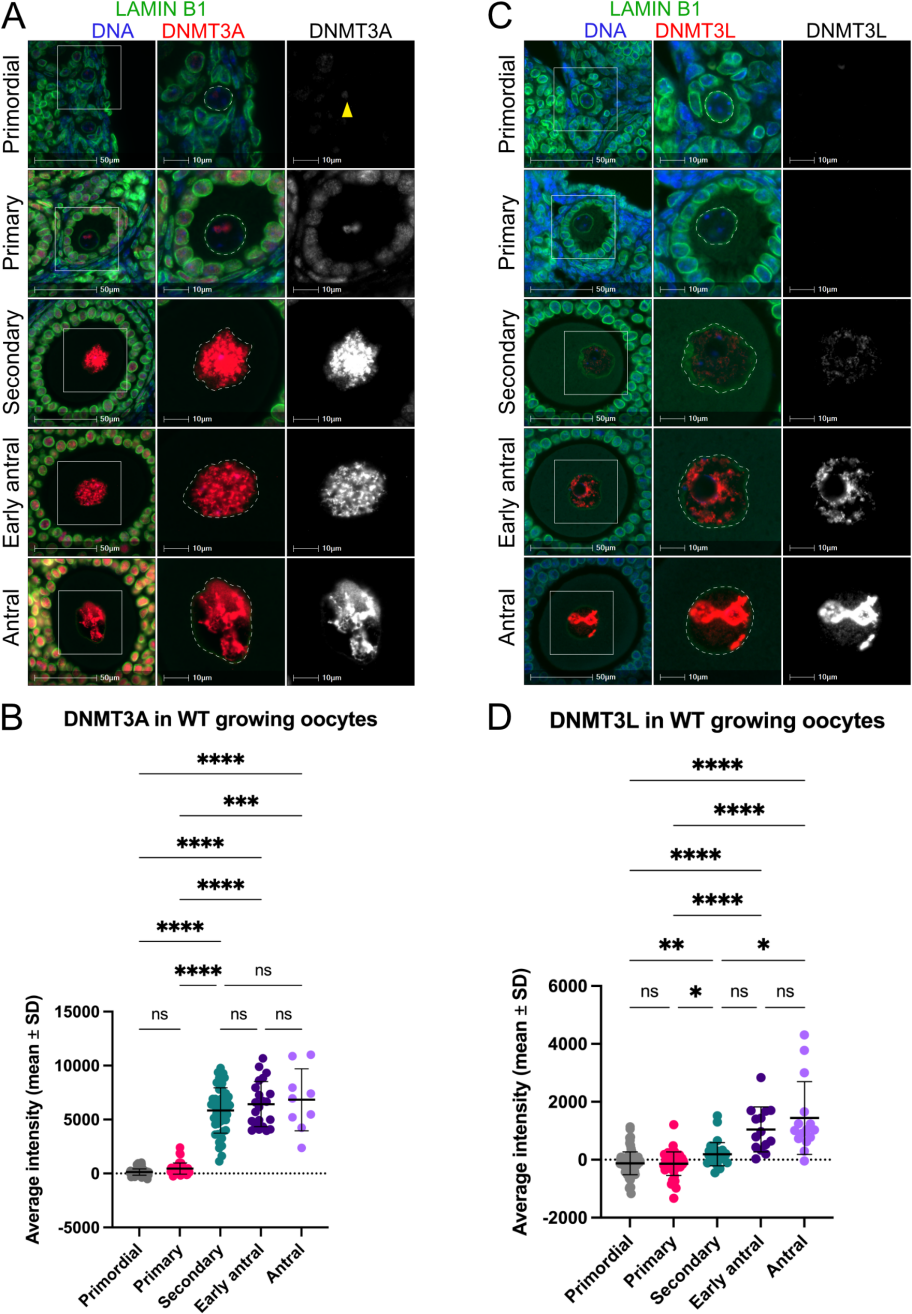
DNMT3A and DNMT3L increase within secondary-antral follicle oocytes. (**A**,** C**) Representative IF images showing DNMT3A (A) and DNMT3L (C) (red, grey single channel) in primordial, primary, secondary, early antral and antral follicle oocytes from wildtype adult mouse ovaries. The oocyte nucleus is defined by Lamin B1 (green, dashed lines), and DNA is shown by DAPI (blue). White boxes indicate regions shown at higher power in images on the right. Yellow arrowhead denotes primordial follicle oocyte nucleus. Images are representative of multiple planes in both ovaries from three biological replicates. Scale bars represent 50 μm (left), and 10 μm (middle, right). **(B**,** D**) Quantification of DNMT3A (B) and DNMT3L (D) within oocyte nuclei of primordial, primary, secondary, early antral and antral follicles from wildtype adult mouse ovaries. Values represent average nuclear staining intensity with average cytoplasmic fluorescence removed to correct for non-specific background staining. Error bars represent mean ± SD. (**B**) ns: not significant, ***P < 0.001, ****P < 0.0001, Kruskal-Wallis with Dunn’s multiple comparisons test. *N* = 86 primordial follicle oocytes, *N* = 45 primary follicle oocytes, *N* = 46 secondary follicle oocytes, *N* = 22 early antral follicle oocytes, and *N* = 9 antral follicle oocytes, in total from three biological replicates. (**D**) ns: not significant, *P < 0.05, **P < 0.01, ****P < 0.0001, Kruskal-Wallis test with Dunn’s multiple comparisons test. *N* = 92 primordial follicle oocytes, *N* = 46 primary follicle oocytes, *N* = 31 secondary follicle oocytes, *N* = 14 early antral follicle oocytes, and *N* = 15 antral follicle oocytes, in total from three biological replicates

The *de novo* DNA methyltransferase DNMT3A and its catalytically inactive co-factor DNMT3L are essential for catalysing *de novo* DNA methylation in growing oocytes [33, 34]. DNMT3A was detected weakly in a single, small faint focal point in the nucleus of primordial follicle stage oocytes, or not at all (Fig. 4A). In primary follicle oocytes, DNMT3A was localised in one to three small circular foci, with minimal staining elsewhere in the nucleus (Fig. 4A). By secondary follicle stage, DNMT3A was broadly distributed throughout the nucleus in a strong punctate pattern, with dramatically increased staining intensity compared to primary follicle oocytes (*P* < 0.0001, Fig. 4A, B). DNMT3A staining remained strong in early antral follicle oocytes, with punctate foci visible broadly throughout the nucleus. In antral follicle oocytes, DNMT3A staining became more localised with large intensely stained regions often associated with the nucleolus and more peripheral regions containing less intense or no staining (Fig. 4A). DNMT3A levels remained consistently high during late oocyte growth, with no significant change in average levels from secondary to antral follicle oocytes (Fig. 4B, Additional File 1: Fig. S1F).

DNMT3L was first observed in secondary follicle oocytes, with no staining detected in primordial or primary follicle oocytes (Fig. 4C). In secondary follicle oocytes, DNMT3L levels increased significantly, and DNMT3L was detected in a weak speckled pattern throughout the nucleus, with some oocytes showing stronger staining than others (*P* < 0.05, Fig. 4C, D). In some early antral follicle oocytes, DNMT3L was detected in a dispersed, speckled pattern throughout the nucleus, while in others, DNMT3L staining was more concentrated in sub-nuclear regions. DNMT3L levels peaked in antral follicle oocytes, with a significant increase from secondary to antral follicle oocytes (*P* < 0.05, Fig. 4D, Additional File 1: Fig. S1G). Antral follicle oocytes were characterised by strong DNMT3L staining in localised sub-nuclear regions (Fig. 4C).

### Loss of EED in growing oocytes increased average SETD2 levels in primary, secondary and early antral follicle oocytes

Given that a previous study found some dependence of H3K27me3 distribution on SETD2 and H3K36me3 [18], we next determined whether KDM6A, RBBP7, DNMT3A, DNMT3L or H3K36me3 levels, or gross nuclear distribution of each differed in oocytes with heterozygous or homozygous *Eed* deletion (*Eed*-het or *Eed*-hom) compared to wildtype *Eed* function (*Eed*-wt). In this model, *Eed* is deleted specifically in oocytes from the primary follicle stage using *Zp3Cre* [10, 35], allowing the impacts of EED loss throughout primary to antral follicle stage oocyte growth, but not primordial follicle stage oocytes, to be determined. Consistent with this, SETD2 levels were not significantly different in primordial follicle oocytes between genotypes (Additional File 1: Fig. S2).

**Fig. 5:**
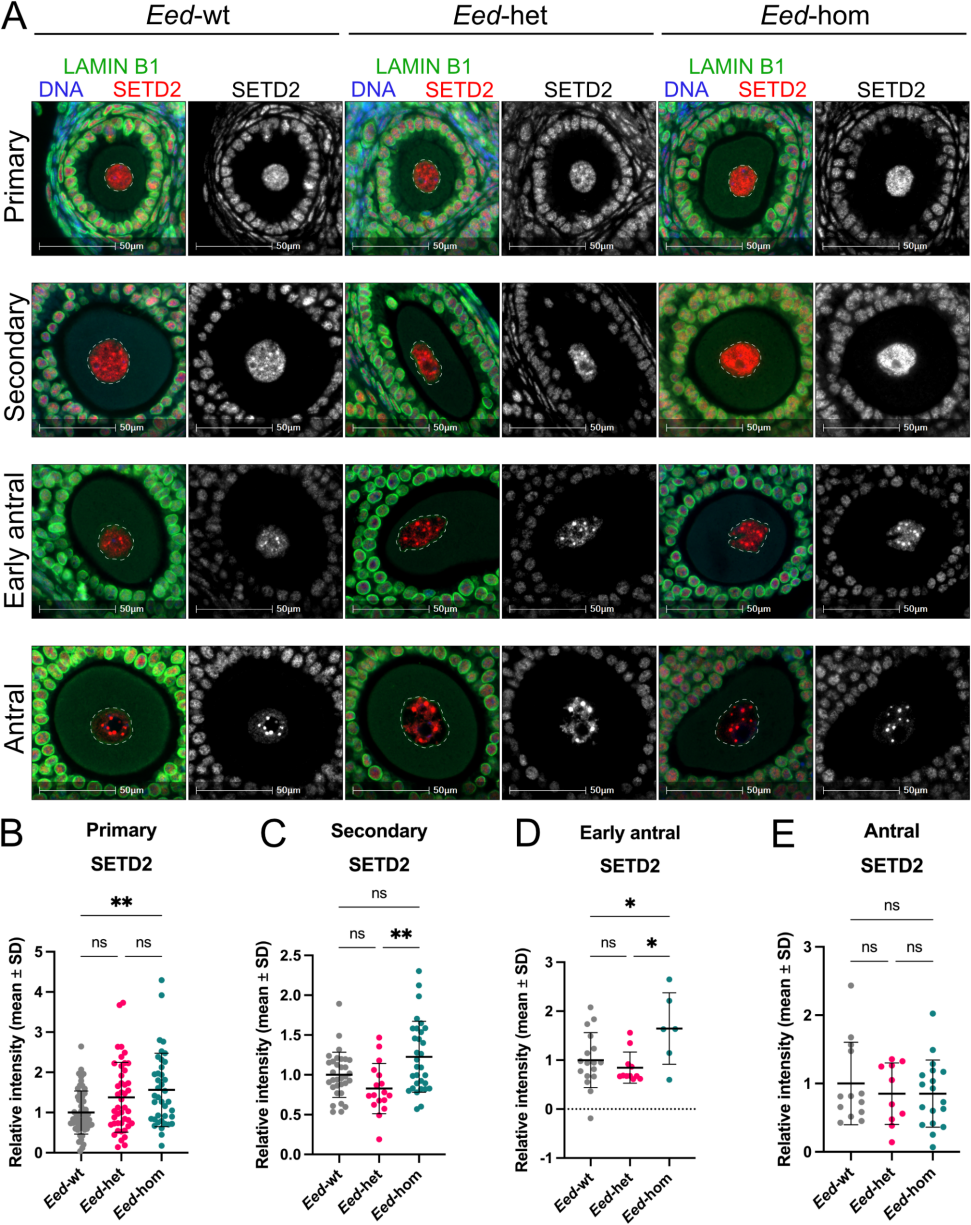
Loss of EED significantly increased SETD2 in primary, secondary, and early antral follicle oocytes. (**A**) Representative IF images showing SETD2 (red, grey single channel) in primary, secondary, early antral and antral follicle oocytes from *Eed*-wt (left), *Eed*-het (middle), and *Eed*-hom (right) adult mouse ovaries. The oocyte nucleus is defined by Lamin B1 (green, dashed lines), and DNA is shown by DAPI (blue). Images are representative of multiple planes in both ovaries from three biological replicates for each genotype. Scale bars represent 50 μm. (**B-E**) Quantification of SETD2 within oocyte nuclei of primary (**B**), secondary (**C**), early antral (**D**), and antral (**E**) follicles from *Eed*-wt, *Eed*-het, and *Eed*-hom adult mouse ovaries. Data is from three biological replicates for each genotype. Values represent average nuclear staining intensity with average cytoplasmic fluorescence removed to correct for non-specific background staining. Average intensity for *Eed*-het and *Eed*-hom samples are shown relative to *Eed*-wt set to 1.0. Error bars represent mean ± SD. (**B-C**) *N* = 56 *Eed*-wt, *N* = 44 *Eed*-het, *N* = 39 *Eed*-hom primary follicle oocytes. *N* = 32 *Eed*-wt, *N* = 17 *Eed*-het, *N* = 31 *Eed*-hom secondary follicle oocytes. ns: not significant, **P < 0.01, Kruskal-Wallis test with Dunn’s multiple comparisons test. (**D-E**) *N* = 17 *Eed*-wt, *N* = 11 *Eed*-het, *N* = 6 *Eed*-hom early antral follicle oocytes. *N* = 13 *Eed*-wt, *N* = 10 *Eed*-het, *N* = 18 *Eed*-hom antral follicle oocytes. ns: not significant, *P < 0.05, one-way ANOVA plus Tukey’s multiple comparisons test

The nuclear staining pattern of SETD2 was similar across *Eed*-wt, *Eed*-het and *Eed*-hom oocytes, with SETD2 initially broadly detected within the nucleus in primordial and primary follicle oocytes and increasingly localised to sub-nuclear regions in later follicle stage oocytes of all three genotypes (Fig. 5A). Notably, quantification revealed that the average nuclear levels of SETD2 were consistently higher in *Eed*-hom primary to early antral follicle stage oocytes compared to *Eed*-wt or *Eed*-het, although this did not reach significance for all comparisons (Fig. 5B-D). By antral follicle stage, SETD2 levels in *Eed*-hom oocytes were similar to *Eed*-het and *Eed*-wt oocytes (Fig. 5E). Overall, while loss of EED and H3K27me3 increased SETD2 levels during early-mid stages of oocyte growth, no differences were observed in SETD2 staining distribution in primary-antral follicle stage oocytes.

### Loss of EED and H3K27me3 in growing oocytes did not impact nuclear levels or localisation of H3K36me3, KDM6A, RBBP7, DNMT3A or DNMT3L

Despite the moderately higher levels of SETD2 observed in *Eed*-hom oocytes, the staining distribution of H3K36me3 was not discernibly affected by loss of H3K27me3 in growing oocytes (Fig. 5, Additional File 1: Fig. S3). H3K36me3 was detected in a speckled pattern throughout the nucleus and was increasingly localised in sub-nuclear regions, particularly around the nucleoli in early antral follicle oocytes, a pattern that was consistent between *Eed*-wt, *Eed*-het and *Eed*-hom oocytes (Additional File 1: Fig. S3). Consistent with the quantification of levels in wild type oocytes (Fig. 1D), H3K36me3 was substantially decreased in oocytes by antral follicle stage (Additional File 1: Fig. S3). KDM6A was also unaffected by loss of EED in oocytes, with diffuse, speckled nuclear staining throughout oocyte growth similar to *Eed*-wt oocytes (Additional File 1: Fig. S4). In all genotypes, KDM6A levels increased in primary-secondary follicle oocytes, and within antral follicle oocytes, KDM6A staining was weaker at regions of the nuclear periphery (Additional File 1: Fig. S4).

Loss of EED in oocytes did not impact RBBP7 nuclear levels, with all genotypes showing speckled staining broadly throughout the nucleus, and a dramatic increase of RBBP7 in secondary follicle oocytes (Additional File 1: Fig. S5). While there were regions of weaker or no RBBP7 staining near the nuclear periphery, and a lack of staining within the nucleolus, these patterns were consistent between *Eed*-wt, *Eed*-het and *Eed*-hom oocytes (Additional File 1: Fig. S5).

DNMT3A distribution was consistent between all genotypes, with small, localised regions of staining in primary follicle oocytes, and substantially increased staining in secondary follicle oocytes (Additional File 1: Fig. S6). Intensity of DNMT3A staining increased in early antral and antral follicle oocytes and was increasingly localised to sub-nuclear regions compared to the secondary stage. In some early antral and antral follicle oocytes, a bright ring of staining was visible surrounding the nucleolus, which was again observed in all genotypes (Additional File 1: Fig. S6). Consistent with observations in *Eed*-wt oocytes, DNMT3L was not detected in *Eed*-het and *Eed*-hom primary follicle oocytes but was visible at low levels in some secondary follicle oocytes (Additional File 1: Fig. S7). In early antral and antral follicle oocytes, DNMT3L staining was also similar between genotypes and characterised by increasingly localised intense sub-nuclear staining as well as regions with weak, diffuse DNMT3L staining (Additional File 1: Fig. S7). Quantification of H3K36me3, KDM6A, RBBP7, DNMT3A and DNMT3L revealed no significant differences in their respective average nuclear levels between *Eed*-wt, *Eed*-het and *Eed*-hom oocytes for the duration of oocyte growth (Additional File 1: Fig. S3-7).

## Discussion

While proper establishment of the oocyte epigenome is essential for normal offspring development, it is unclear how different epigenetic complexes interact to establish their respective modifications within growing oocytes, particularly in adult ovaries. Kageyama and colleagues provided a detailed assessment of several acetylation modifications, methylation of H3K4 and H3K9 and enrichment of 5-methyl-cytosine in individual growing oocytes during the first wave of oocyte growth at postnatal day (P) 5, P10, P15 and GV stages [36]. In addition, PRC2 is transiently expressed in primary-secondary follicle oocytes, when all three essential core components (EED, EZH2, and SUZ12) are present within the nucleus [20]. However, detailed studies of histone modifications and histone modifying proteins throughout the growth period of oocytes have been rare and have not detailed the spatiotemporal distribution of key modifiers and modifications in the context of growing follicles in adult ovaries.

This study characterised the spatial and temporal profiles of SETD2, KDM6A, RBBP7, DNMT3A and DNMT3L, as well as changes in global distribution and levels of H3K36me3 and H3K27me3 in growing oocytes. These analyses demonstrate that reorganisation of histone modifications by histone methyltransferases and demethylases likely occurs in early oocyte growth, preceding *de novo* DNA methylation. This implies that a highly ordered process of oocyte epigenetic programming is required to ultimately establish the fully developed and distinct epigenome of fully grown oocytes. Whilst this work provides insight into mouse oocyte epigenetic programming, studies in human growing oocytes are limited for comparison. Several studies have analysed transcript levels of PRC2 subunits and other epigenetic regulators in human growing oocytes or follicles, or fully grown and mature oocytes [37–40]. Moreover, evidence suggests that many of the genes marked by H3K27me3 and de-repressed after loss of EED in mouse oocytes are also marked by H3K27me3 in human GV stage oocytes, suggesting conservation of this mechanism [20, 41]. In another study IF was used to detect several epigenetic modifiers and modifications in human growing oocytes, but this study did not reveal their temporal organisation throughout different stages of oocyte growth [40]. Further analysis of temporal and spatial regulation of epigenetic modifiers and modifications in human growing oocytes throughout folliculogenesis is an area that requires further research.

RBBP7 associates with EED, EZH2, and SUZ12, forming a stable, tetrameric core which mediates establishment of H3K27me3 [21]. Our results show that RBBP7 increases substantially in secondary follicle oocytes, during the time when EED, EZH2, and SUZ12 are transiently detected together in growing oocytes and establish H3K27me3 on developmental gene promoters [20]. Additionally, our results demonstrate that SETD2 increased in primary-secondary follicle oocytes, during the window in which EED and SUZ12 were also detected with EZH2 and RBBP7 [20]. This suggests that SETD2 and PRC2 respectively deposit H3K36me3 and H3K27me3 with similar timing in primary-secondary follicle oocytes. Increasing localisation of SETD2 in later oocyte growth may represent discrete chromatin regions where SETD2 is recruited to deposit H3K36me3. H3K36me3 marks chromatin accessible for transcription in oocytes [18], such that SETD2/H3K36me3 foci would be localised near regions of active transcription. Despite H3K36me3 levels not increasing at the same time, the increased SETD2 level in primary-secondary follicle oocytes may be explained by a role for SETD2 in remodelling of H3K36me3 without changing overall levels. This is likely to include genes that gain H3K36me3 and expression but lose H3K27me3 between P7 and P14 in growing oocytes [18].

The patterns of H3K36me3 sub-nuclear localisation are quite dynamic. Initially as oocytes grow though secondary and early antral stages H3K36me3 is increasingly localised in sub-nuclear foci, perhaps reflecting the separation of H3K36me3-enriched chromatin from repressed chromatin domains. However, at late antral stages there was a precipitous decrease in H3K36me3 staining. As H3K36me3 marks transcribed regions and fully grown oocytes progress from transcriptionally active non-surrounded nucleolus (NSN) GV oocytes to transcriptionally silent SN GV oocytes [42, 43], it appears likely that the global loss of H3K36me3 reflects the shift of oocytes into their transcriptionally inactive stage prior to maturation.

In contrast to H3K36me3 which marks active transcription, H3K27me3 is required for gene repression. As was described for both SETD2 and H3K36me3, increased H3K27me3 localisation to specific sub-nuclear regions in late oocyte growth likely reflects changes in H3K27me3-enriched chromatin organisation. H3K27me3 levels transiently increased in primary follicle oocytes, which coincides with transient expression of EZH2, EED and SUZ12 and H3K27me3 establishment on the promoters of developmental genes in primary-secondary follicle oocytes [20]. Subsequently, H3K27me3 decreased in secondary follicle oocytes, potentially indicating further remodelling of H3K27me3. Notably, this corresponded with increased KDM6A in late primary to secondary follicle oocytes that directly preceded the decrease in H3K27me3 observed in secondary follicle oocytes, consistent with KDM6A demethylation of H3K27. This is likely to include genes that preferentially lose H3K27me3 from P14 onwards in growing oocytes [19].

Previous studies have demonstrated the dependence of *de novo* DNA methylation on H3K36me3 establishment in growing oocytes [18, 44, 45]. However, despite previous observations that *Dnmt3a* and *Dnmt3l* are transcribed, and DNA methylation is established in oocytes of antral stage follicles, the protein distribution of DNMT3A and DNMT3L in growing oocytes was unknown. The IF data shown here reveal that both DNMT3A and DNMT3L proteins rapidly increase in growing oocytes from the secondary stage. This is consistent with *Dnmt3a* and *Dnmt3l* expression levels increasing with oocyte diameter, with peak expression in fully grown oocytes [46]. While DNMT3A was detected in very small, discrete foci in oocytes of primary and to a lesser extent some primordial follicles, the significance of this staining remains unknown. Notwithstanding this qualification, the observations that both PRC2 activity and peak SETD2 levels in primary-secondary follicle oocytes precede DNMT3A and DNMT3L in secondary-antral follicle oocytes are consistent with the establishment and remodelling of histone modifications prior to *de novo* DNA methylation.

SETD2 and H3K36me3 are required for normal establishment of DNA methylation. Moreover, loss of H3K36me3 leads to the expansion of H3K27me3 within specific oocyte domains, but neither H3K27me3 nor H3K36me3 are altered by deletion of *Dnmt3l* [18]. In addition, while loss of SETD2 profoundly affected gene expression, H3K36me3 and DNA methylation in fully grown oocytes, loss of SETD2 did not affect gene transcription or H3K27me3 distribution in oocytes of primary follicles isolated from P7 ovaries [18]. Together, these observations suggest that H3K36me3 and H3K27me3 are both established and removed by their respective epigenetic modifiers in primary-secondary follicle oocytes to create a histone ‘template’ that subsequently directs DNA methylation and possibly other epigenetic modifications. This is not only important for the proper establishment of canonical and non-canonical (H3K27me3) imprints but is likely also important for the regulation of the epigenome more broadly during oocyte growth.

In addition to the temporal changes in levels of epigenetic modifiers and modifications examined in this study, the spatial patterns observed using IF were also of interest. In early antral and antral follicle oocytes, DNMT3A and DNMT3L were localised in a ring around the nucleolus, consistent with their co-localisation with heterochromatin in SN GV oocytes [43, 47]. Although the significance of the small foci of DNMT3A staining detected in primordial and primary follicle oocytes is unclear, it was evident that these foci occurred in the absence of any detectable DNMT3L. It was also evident that DNMT3L was substantially weaker than DNMT3A in secondary follicle oocytes and did not reach its peak until antral stages. DNMT3A facilitates *de novo* DNA methylation in somatic cells without DNMT3L, and it has also been suggested that DNA methylation in oocytes can be dependent or independent of DNMT3L [48, 49]. This raises the possibility that specific sequences may be methylated by DNMT3A early in oocyte growth before DNMT3L is detected, although it is unclear which sequences may be targeted. One study has suggested that more highly expressed gene bodies gain methylation earlier in growing oocytes [50], which may be DNMT3A-mediated. However, further research is necessary to identify which sequences DNMT3A may methylate independent of DNMT3L during early oocyte growth. In later oocyte growth following histone modification reorganisation when both DNMT3A and DNMT3L are detected, methylation of sequences which require both DNMTs is expected to occur. For example, DNMT3A and DNMT3L are together required for CpG methylation including maternal imprints [33, 51]. Consistent with this, CpG islands (CGIs) acquire methylation on average later than the rest of the genome [50]. In addition, later methylated CGIs are more dependent on removal of H3K4me3 [17, 50], which further emphasises the importance of histone modification remodelling to ensure appropriate DNA methylation.

It has been proposed that PRC2 establishment of H3K27me3 on developmental gene promoters in primary-secondary follicle oocytes may prevent the establishment of other epigenetic modifications during oocyte growth [20]. Complete loss of EED resulted in a reduction of 35% of H3K27me3 in primary follicle oocytes, and 85% in secondary follicle oocytes, while heterozygous *Eed* deletion maintained similar H3K27me3 levels [20]. As KDM6A levels were unchanged by loss of EED, and passive demethylation is unlikely to be facilitated as growing oocytes are not dividing [44], it appears likely that KDM6A (and/or KDM6B) mediates the rapid reduction in H3K27me3 observed in *Eed*-hom primary-secondary follicle oocytes.

Loss of SETD2 in growing oocytes depletes H3K36me3 and leads to ectopic establishment of H3K27me3 in a highly region-specific manner [18]. While we found that loss of EED and H3K27me3 increased SETD2 levels indicating some impact of EED loss on H3K36me3, another study indicated that loss of EED and H3K27me3 did not affect H3K36me3 distribution in fully grown oocytes [38]. The latter observation indicates that loss of EED and H3K27me3 is likely to have a less striking impact on H3K36me3 localisation than the H3K27me3 spreading caused by deletion of *Setd2* [18]. However, distribution of H3K27me3 and H3K36me3 was examined in fully grown oocytes in the *Setd2* deletion model and loss of EED increased SETD2 levels in primary-early antral follicle stage oocytes, but not in antral follicle oocytes. It therefore remains possible that loss of EED causes alterations in H3K36me3 in primary-secondary follicle stage oocytes that were not observable via H3K36me3 distribution in fully grown oocytes. Moreover, given that an H3K27me3 - H3K36me3 template appears to be established in primary-secondary follicle oocytes and *de novo* DNA methylation follows the H3K36me3 template [18], it is perhaps more likely that loss of H3K27me3 alters DNA methylation rather than H3K36me3 distribution in fully grown oocytes.

Finally, while EZH2, EED and SUZ12 are all transiently detected together in primary-secondary follicle oocytes during which H3K27me3 is established on developmental genes [20], RBBP7 remained strongly detected in the nucleus of early antral and antral follicle oocytes in the absence of EED and SUZ12 in wildtype oocytes and was unaffected by deletion of *Eed*. This is intriguing as it indicates that RBBP7 localisation within the nucleus is not dependent on EED and that RBBP7 is likely to have PRC2 independent functions within growing oocytes. We observed a similar pattern for EZH2 [20], highlighting the possibility that there is significant variation in the temporal and spatial profiles for important binding partners that are essential for PRC2 function. Notably, an earlier study proposed a PRC2 independent role for EZH2 in meiosis [52] and a recent study revealed this role in more detail [53]. Together these observations highlight a greater need to understand domains within these co-factors that may regulate processes outside the PRC2-dependent methylation of H3K27.

## Conclusions

In this study we provide detailed spatial and temporal profiles of epigenetic modifiers and modifications particularly associated with H3K27me3, H3K36me3 and DNA methylation in growing oocytes of primordial to antral follicles. Together with published data, our immunofluorescence data are consistent with a critical histone remodelling event in primary-secondary follicle oocytes that precedes *de novo* DNA methylation in secondary to antral follicle oocytes. This appears to result in the establishment of an interdependent histone modification template that directs the establishment of DNA methylation at appropriate loci and draws particular attention to a highly ordered process that underpins oocyte epigenetic programming. Together, these studies provide increasing insight into the importance of tightly controlled regulation of epigenetic modifiers as they deposit their respective modifications to regulate establishment of the oocyte epigenome. As disruptions to the oocyte epigenome can disrupt epigenetic memory and alter developmental outcomes in the next generation, extending our knowledge of the complexity and interdependence of oocyte epigenetic programming factors and their potential impacts on maternal epigenetic inheritance is essential for fully understanding inherited disorders.

## Electronic supplementary material

Below is the link to the electronic supplementary material.

Supplementary Material 1

## Data Availability

Sequence data that support the findings of this study have been deposited in the Gene Expression Omnibus archive with the GEO accession number GSE193582.

## Abbreviations

CGI: CpG island
DNMT3A/3L: DNA methyltransferase 3 alpha or 3 like
EED: Embryonic Ectoderm Development
EZH1/2: Enhancer of Zeste 1 or 2
GV: Germinal vesicle
H3K27me3: Histone 3 Lysine 27 trimethylation
H3K36me3: Histone 3 Lysine 36 trimethylation
KDM6A: Lysine demethylase 6A
NSN: Non-surrounded nucleolus
PRC2: Polycomb Repressive Complex 2
RBBP4/7: Retinoblastoma Binding Protein 4 or 7
SETD2: SET domain containing 2, histone lysine methyltransferase
Sfmbt2: Scm like with fut mbt domains 2
Slc38a4: Solute carrier family 38 member 4
SN: Surrounded nucleolus
SUZ12: Suppressor of Zeste 12
Xist: X-inactive specific transcript
