# Supplementary material for: Coordinated regulation of chromatin modifiers reflects organised epigenetic programming in mouse oocytes
Source: Epigenetics Chromatin. 2025 Apr 5;18:19. doi: 10.1186/s13072-025-00583-9 (PMC11971813; doi:10.1186/s13072-025-00583-9)
Supplement: Supplementary file 1 — Supplementary Material 1 [file 13072_2025_583_MOESM1_ESM.pdf]

Additional File 1

Coordinated regulation of chromatin modifiers reflects organised epigenetic programming in mouse oocytes

Authors: Chloe A. Edwards-Lee[1], Ellen G. Jarred[1] and Patrick S. Western[1]

Affiliations: [1]Centre for Reproductive Health, Hudson Institute of Medical Research and Department of Molecular and Translational Science, Monash University, Clayton, Victoria, Australia.

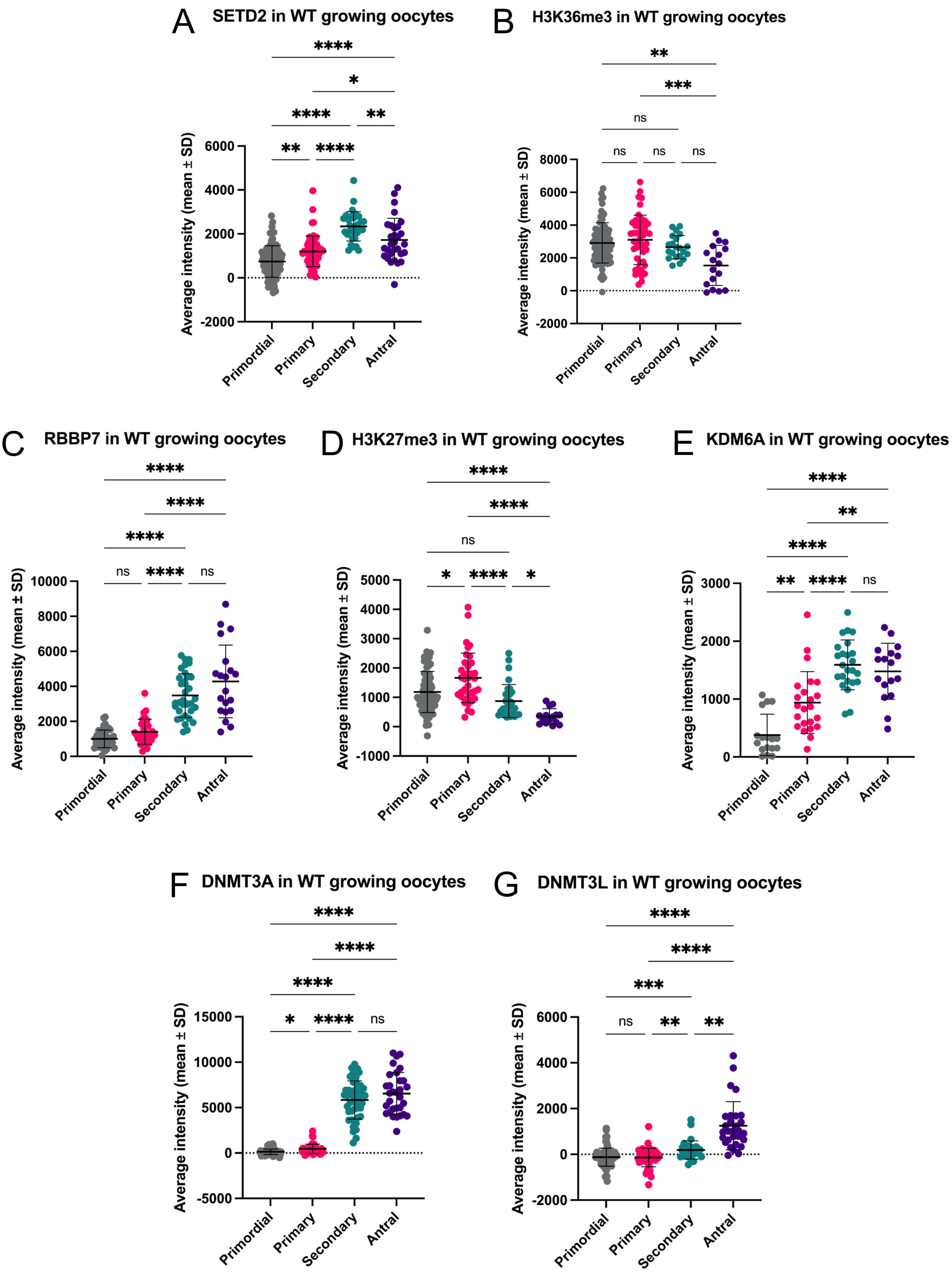
A
SETD2 in WT growing oocytes
****
*
****
**
**
****
6000
4000
2000
0
-2000
Average intensity (mean ± SD)
Primordial
Primary
Secondary
Antral
B
H3K36me3 in WT growing oocytes
**
***
ns
ns
ns
ns
8000
6000
4000
2000
0
-2000
Average intensity (mean ± SD)
Primordial
Primary
Secondary
Antral
C
RBBP7 in WT growing oocytes
****
****
****
ns
****
ns
10000
8000
6000
4000
2000
0
Average intensity (mean ± SD)
Primordial
Primary
Secondary
Antral
D
H3K27me3 in WT growing oocytes
****
****
ns
*
****
*
5000
4000
3000
2000
1000
0
-1000
Average intensity (mean ± SD)
Primordial
Primary
Secondary
Antral
E
KDM6A in WT growing oocytes
****
**
****
**
****
ns
3000
2000
1000
0
Average intensity (mean ± SD)
Primordial
Primary
Secondary
Antral
F
DNMT3A in WT growing oocytes
****
****
****
*
****
ns
15000
10000
5000
0
-5000
Average intensity (mean ± SD)
Primordial
Primary
Secondary
Antral
G
DNMT3L in WT growing oocytes
****
****
***
ns
**
**
6000
4000
2000
0
-2000
Average intensity (mean ± SD)
Primordial
Primary
Secondary
Antral

Figure S1. Assessment of the average staining intensity of epigenetic modifiers and modifications considering early and advanced antral follicles together. Quantification of SETD2 (A), H3K36me3 (B), RBBP7 (C), H3K27me3 (D), KDM6A (E), DNMT3A (F) and DNMT3L (G) within oocyte nuclei of primordial, primary, secondary, and antral follicles from wildtype adult mouse ovaries. Values represent average nuclear staining intensity with average cytoplasmic fluorescence removed to correct for non-specific background staining. Error bars represent mean ± SD. Number of oocytes assessed at each stage in primordial, primary, secondary and antral follicle oocytes: **(A)** N=94 primordial, N=56 primary; N=32 secondary; N=30 antral; three biological replicates. **(B)** N=82 primordial; N=47 primary; N=21 secondary; N=17 antral; three biological replicates. **(C)** N=41 primordial; N=30 primary; N=35 secondary; N=20 antral; two biological replicates. **(D)** N=84 primordial; N=37 primary; N=32 secondary; N=15 antral; three biological replicates. **(E)** N=17 primordial; N=23 primary; N=24 secondary; N=17 antral; two biological replicates. **(F)** N=86 primordial; N=45 primary; N=46 secondary; N=31 antral; three biological replicates. **(G)** N=92 primordial; N=46 primary; N=31 secondary; N=29 antral; three biological replicates. **(A, E)** ns: not significant, *P<0.05, **P<0.01, ****P<0.0001, one-way ANOVA plus Tukey's multiple comparisons test, **(B, C, D, F, G)** ns: not significant, *P<0.05, **P<0.01, ***P<0.001, ****P<0.0001, Kruskal-Wallis test with Dunn's multiple comparisons test.

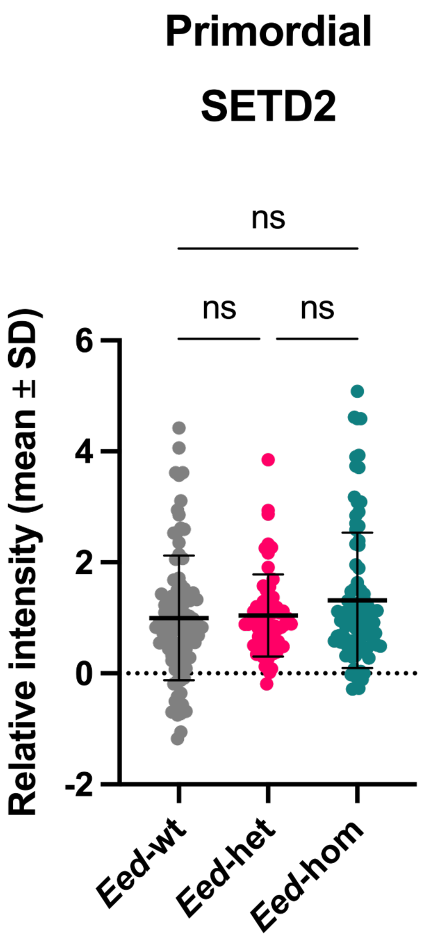

Primordial
SETD2
ns
ns
ns
Relative intensity (mean ± SD)
6
4
2
0
-2
Eed-wt
Eed-het
Eed-hom

Figure S2. SETD2 levels in primordial follicle oocytes were similar between genotypes before *Zp3Cre*-mediated *Eed* deletion. Quantification of SETD2 within oocyte nuclei of primordial follicles from *Eed*-wt, *Eed*-het, and *Eed*-hom adult mouse ovaries. Values represent average nuclear staining intensity with average cytoplasmic fluorescence removed to correct for non-specific background staining. Average intensity for *Eed*-het and *Eed*-hom samples are shown relative to *Eed*-wt set to 1.0. Error bars represent mean ± SD. Three biological replicates with the following number of primordial follicle oocytes assessed: *Eed*-wt N=94, *Eed*-het N=63, *Eed*-hom N=91. ns: not significant, Kruskal-Wallis test with Dunn's multiple comparisons test.

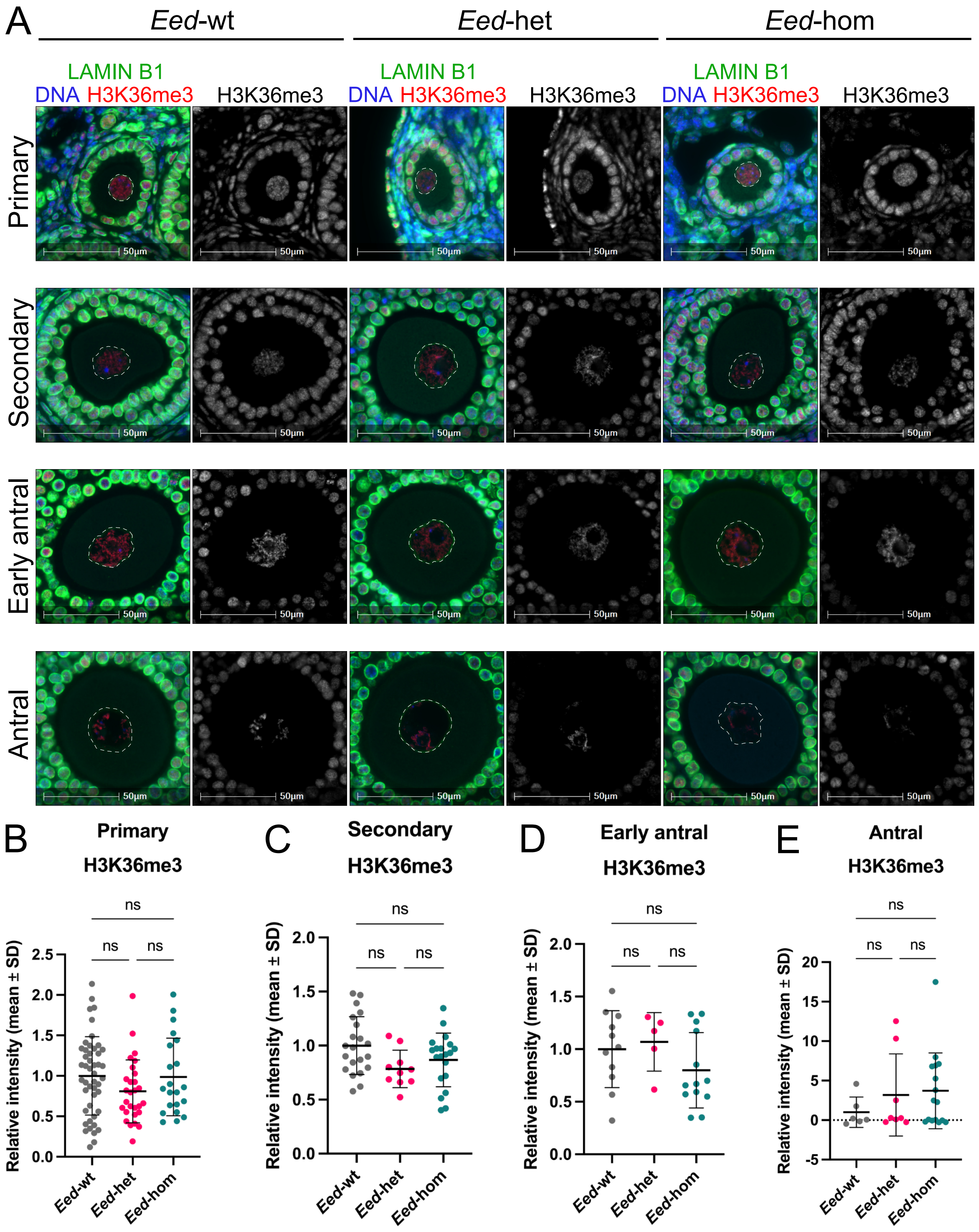
A
Eed-wt
Eed-het
Eed-hom
LAMIN B1
DNA H3K36me3
H3K36me3
Primary
Secondary
Early antral
Antral
50µm
B
Primary
H3K36me3
C
Secondary
H3K36me3
D
Early antral
H3K36me3
E
Antral
H3K36me3
ns
Relative intensity (mean ± SD)
Eed-wt
Eed-het
Eed-hom

Figure S3. Average H3K36me3 levels were unchanged by loss of H3K27me3 in growing oocytes. (A) Representative IF images showing H3K36me3 (red, grey single channel) in primary, secondary, early antral and antral follicle oocytes from *Eed*-wt (left), *Eed*-het (middle), and *Eed*-hom (right) adult mouse ovaries. The oocyte nucleus is defined by Lamin B1 (green), and DNA is shown by DAPI (blue). Images are representative of multiple planes in both ovaries from three biological replicates for each genotype. Scale bars represent 50µm. **(B-E)** Quantification of H3K36me3 within oocyte nuclei of primary **(B)**, secondary **(C)**, early antral **(D)**, and antral **(E)** follicles from *Eed*-wt, *Eed*-het, and *Eed*-hom adult mouse ovaries. Data is from three biological replicates for each genotype. Average intensity for *Eed*-het and *Eed*-hom samples are shown relative to *Eed*-wt set to 1.0. Error bars represent mean ± SD. N=47 *Eed*-wt, N=28 *Eed*-het, N=21 *Eed*-hom primary follicle oocytes. N=21 *Eed*-wt, N=10 *Eed*-het, N=20 *Eed*-hom secondary follicle oocytes. N=11 *Eed*-wt, N=5 *Eed*-het, N=13 *Eed*-hom early antral follicle oocytes. N=6 *Eed*-wt, N=8 *Eed*-het, N=16 *Eed*-hom antral follicle oocytes. ns: not significant, one-way ANOVA plus Tukey's multiple comparisons test.

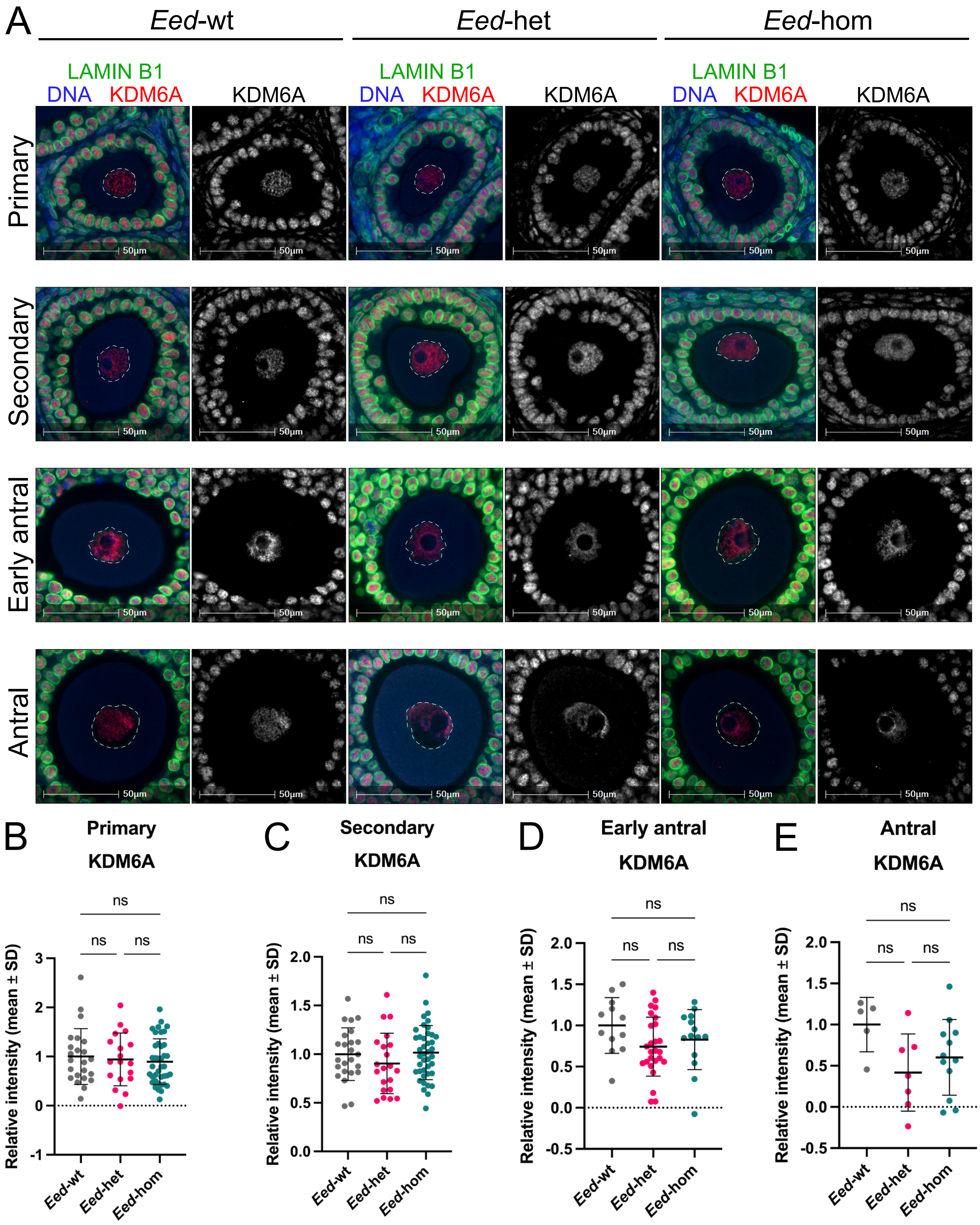
A
Eed-wt
Eed-het
Eed-hom
LAMIN B1
DNA KDM6A
KDM6A
Primary
Secondary
Early antral
Antral
50µm
B
Primary
KDM6A
C
Secondary
KDM6A
D
Early antral
KDM6A
E
Antral
KDM6A
ns
Relative intensity (mean ± SD)
Eed-wt
Eed-het
Eed-hom

Figure S4. Loss of EED and H3K27me3 did not impact KDM6A levels throughout oocyte growth. (A) Representative IF images showing KDM6A (red, grey single channel) in primary, secondary, early antral and antral follicle oocytes from *Eed*-wt (left), *Eed*-het (middle), and *Eed*-hom (right) adult mouse ovaries. The oocyte nucleus is defined by Lamin B1 (green), and DNA is shown by DAPI (blue). Images are representative of multiple planes in both ovaries from two biological replicates for each genotype. Scale bars represent 50µm. **(B-E)** Quantification of KDM6A within oocyte nuclei of primary **(B)**, secondary **(C)**, early antral **(D)**, and antral **(E)** follicles from *Eed*-wt, *Eed*-het, and *Eed*-hom adult mouse ovaries. Data is from two biological replicates for each genotype. Average intensity for *Eed*-het and *Eed*-hom samples are shown relative to *Eed*-wt set to 1.0. Error bars represent mean ± SD. N=23 *Eed*-wt, N=18 *Eed*-het, N=39 *Eed*-hom primary follicle oocytes. N=24 *Eed*-wt, N=21 *Eed*-het, N=41 *Eed*-hom secondary follicle oocytes. N=12 *Eed*-wt, N=26 *Eed*-het, N=14 *Eed*-hom early antral follicle oocytes. N=5 *Eed*-wt, N=7 *Eed*-het, N=12 *Eed*-hom antral follicle oocytes. ns: not significant, one-way ANOVA plus Tukey's multiple comparisons test.

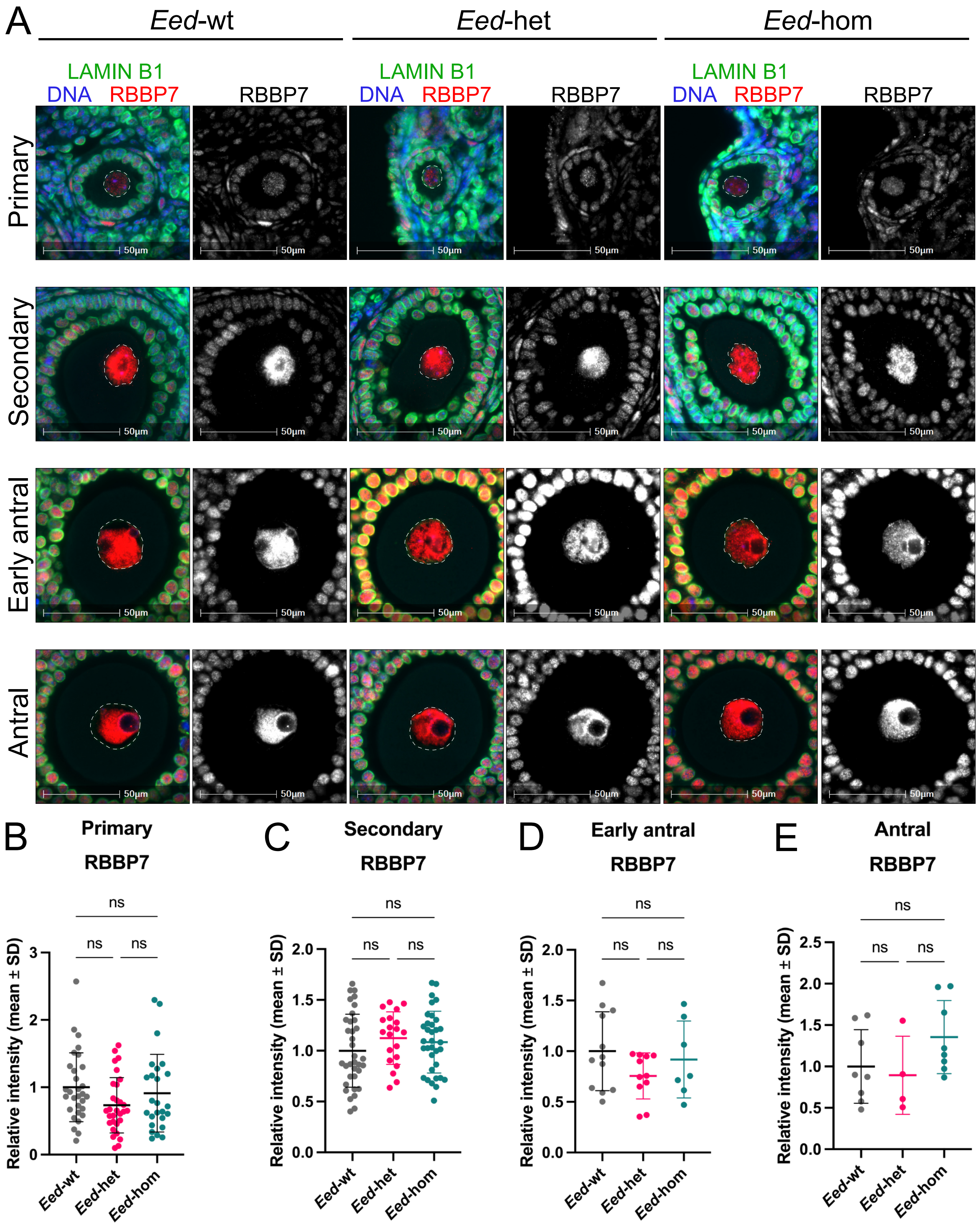
A
Eed-wt
Eed-het
Eed-hom
LAMIN B1
DNA RBBP7
RBBP7
Primary
Secondary
Early antral
Antral
50µm
B
Primary
RBBP7
C
Secondary
RBBP7
D
Early antral
RBBP7
E
Antral
RBBP7
ns
Relative intensity (mean ± SD)
Eed-wt
Eed-het
Eed-hom

Figure S5. Average RBBP7 levels were not significantly different between *Eed*-wt, *Eed*-het, and *Eed*-hom growing oocytes. (A) Representative IF images showing RBBP7 (red, grey single channel) in primary, secondary, early antral and antral follicle oocytes from *Eed*-wt (left), *Eed*-het (middle), and *Eed*-hom (right) adult mouse ovaries. The oocyte nucleus is defined by Lamin B1 (green), and DNA is shown by DAPI (blue). Images are representative of multiple planes in both ovaries from two biological replicates for each genotype. Scale bars represent 50µm. **(B-E)** Quantification of RBBP7 within oocyte nuclei of primary **(B)**, secondary **(C)**, early antral **(D)**, and antral **(E)** follicles from *Eed*-wt, *Eed*-het, and *Eed*-hom adult mouse ovaries. Data is from two biological replicates for each genotype. Average intensity for *Eed*-het and *Eed*-hom samples are shown relative to *Eed*-wt set to 1.0. Error bars represent mean ± SD. N=30 *Eed*-wt, N=30 *Eed*-het, N=25 *Eed*-hom primary follicle oocytes. N=35 *Eed*-wt, N=20 *Eed*-het, N=34 *Eed*-hom secondary follicle oocytes. N=12 *Eed*-wt, N=11 *Eed*-het, N=7 *Eed*-hom early antral follicle oocytes. N=8 *Eed*-wt, N=4 *Eed*-het, N=8 *Eed*-hom antral follicle oocytes. ns: not significant, one-way ANOVA plus Tukey's multiple comparisons test.

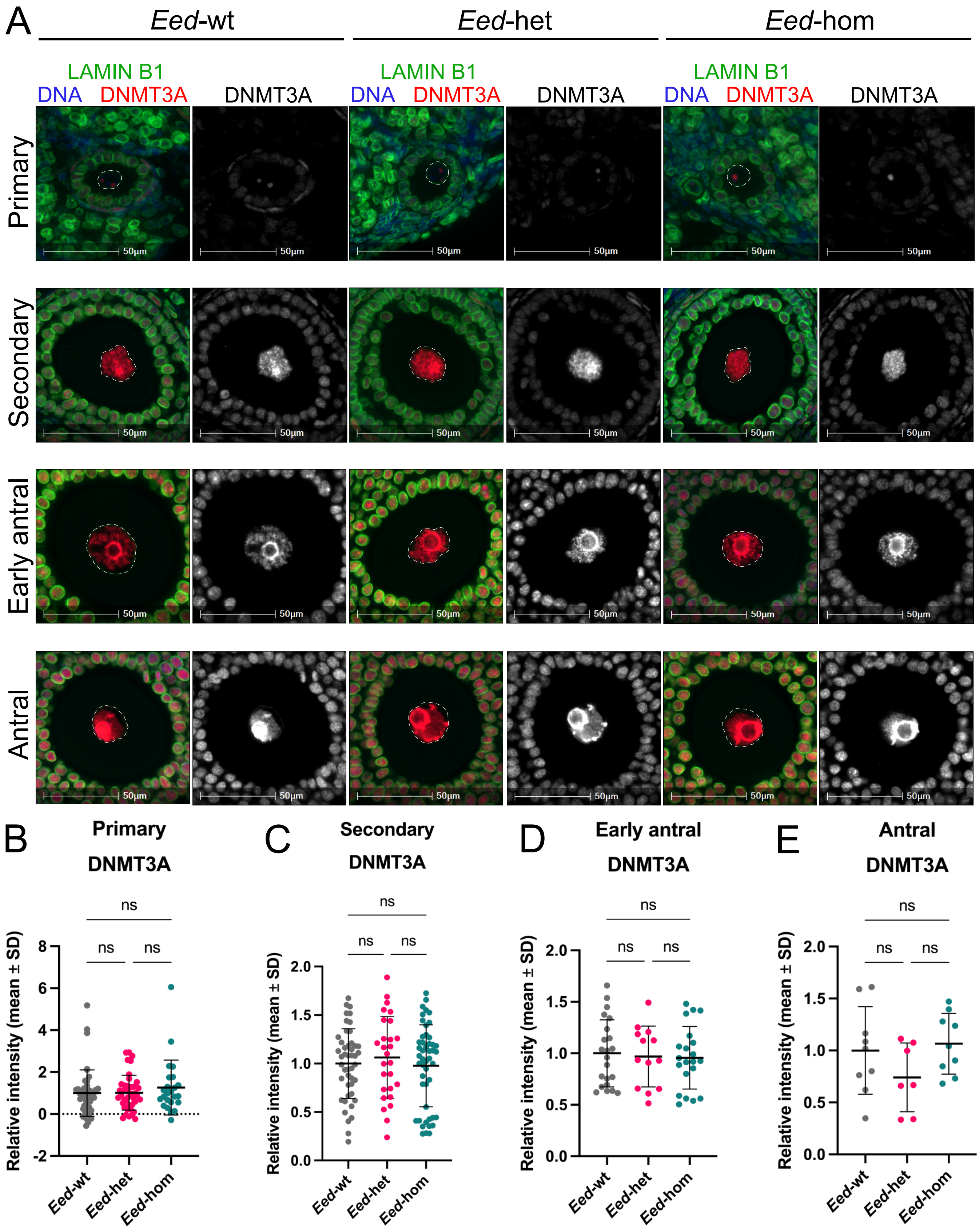
A
Eed-wt
Eed-het
Eed-hom
LAMIN B1
DNA DNMT3A
DNMT3A
Primary
Secondary
Early antral
Antral
50μm
B
Primary
DNMT3A
C
Secondary
DNMT3A
D
Early antral
DNMT3A
E
Antral
DNMT3A
ns
Relative intensity (mean ± SD)
Eed-wt
Eed-het
Eed-hom

Figure S6. Loss of EED did not impact DNMT3A levels in growing oocytes. (A) Representative IF images showing DNMT3A (red, grey single channel) in primary, secondary, early antral and antral follicle oocytes from *Eed*-wt (left), *Eed*-het (middle), and *Eed*-hom (right) adult mouse ovaries. The oocyte nucleus is defined by Lamin B1 (green), and DNA is shown by DAPI (blue). Images are representative of multiple planes in both ovaries from three biological replicates for each genotype. Scale bars represent 50µm. **(B-E)** Quantification of DNMT3A within oocyte nuclei of primary **(B)**, secondary **(C)**, early antral **(D)**, and antral **(E)** follicles from *Eed*-wt, *Eed*-het, and *Eed*-hom adult mouse ovaries. Data is from three biological replicates for each genotype. Average intensity for *Eed*-het and *Eed*-hom samples are shown relative to *Eed*-wt set to 1.0. Error bars represent mean ± SD. **(B)** N=45 *Eed*-wt, N=42 *Eed*-het, N=24 *Eed*-hom primary follicle oocytes. ns: not significant, Kruskal-Wallis test with Dunn's multiple comparisons test. **(C-E)** N=46 *Eed*-wt, N=27 *Eed*-het, N=48 *Eed*-hom secondary follicle oocytes. N=22 *Eed*-wt, N=13 *Eed*-het, N=21 *Eed*-hom early antral follicle oocytes. N=9 *Eed*-wt, N=7 *Eed*-het, N=9 *Eed*-hom antral follicle oocytes. ns: not significant, one-way ANOVA plus Tukey's multiple comparisons test.

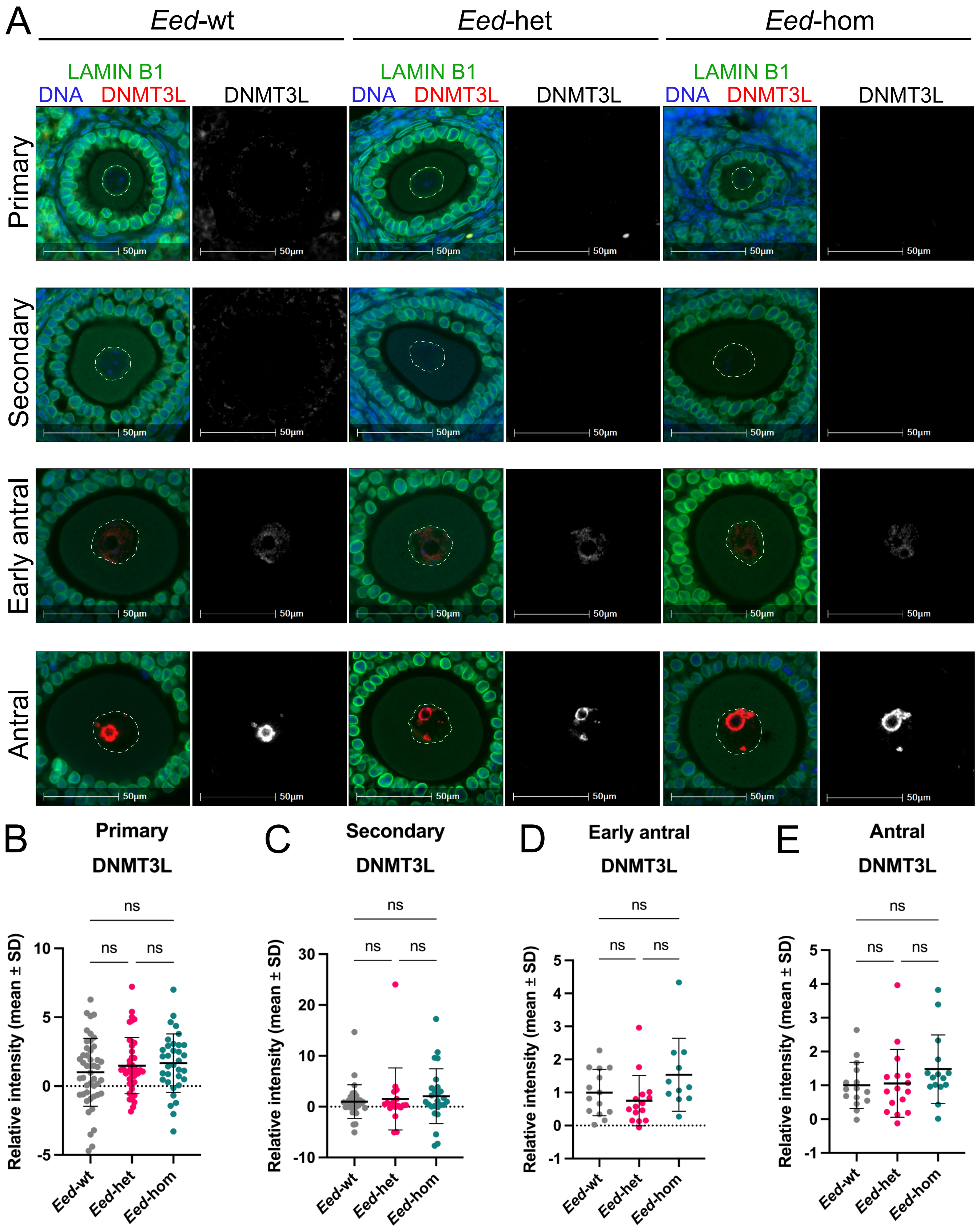

A
Eed-wt
Eed-het
Eed-hom
LAMIN B1
DNA DNMT3L
DNMT3L
Primary
Secondary
Early antral
Antral
50µm
B
Primary
DNMT3L
C
Secondary
DNMT3L
D
Early antral
DNMT3L
E
Antral
DNMT3L
ns
Relative intensity (mean ± SD)
Eed-wt
Eed-het
Eed-hom

Figure S7. Loss of EED did not impact DNMT3L levels in growing oocytes. (A) Representative IF images showing DNMT3L (red, grey single channel) in primary, secondary, early antral and antral follicle oocytes from *Eed*-wt (left), *Eed*-het (middle), and *Eed*-hom (right) adult mouse ovaries. The oocyte nucleus is defined by Lamin B1 (green), and DNA is shown by DAPI (blue). Images are representative of multiple planes in both ovaries from three biological replicates for each genotype. Scale bars represent 50µm. **(B-E)** Quantification of DNMT3L within oocyte nuclei of primary **(B)**, secondary **(C)**, early antral **(D)**, and antral **(E)** follicles from *Eed*-wt, *Eed*-het, and *Eed*-hom adult mouse ovaries. Data is from three biological replicates for each genotype. Average intensity for *Eed*-het and *Eed*-hom samples are shown relative to *Eed*-wt set to 1.0. Error bars represent mean ± SD. **(B, D, E)** N=46 *Eed*-wt, N=42 *Eed*-het, N=34 *Eed*-hom primary follicle oocytes. N=14 *Eed*-wt, N=15 *Eed*-het, N=11 *Eed*-hom early antral follicle oocytes. N=15 *Eed*-wt, N=16 *Eed*-het, N=15 *Eed*-hom antral follicle oocytes. ns: not significant, one-way ANOVA plus Tukey's multiple comparisons test. **(C)** N=31 *Eed*-wt, N=18 *Eed*-het, N=26 *Eed*-hom secondary follicle oocytes. ns: not significant, Kruskal-Wallis test with Dunn's multiple comparisons test.
